# Structural, Thermodynamic, and Spectroscopic Characterization of Diphosgene and Triphosgene

**DOI:** 10.1021/acs.inorgchem.5c05882

**Published:** 2026-03-20

**Authors:** Sven Ringelband, Stewart F. Parker, Frank Tambornino

**Affiliations:** † Department of Chemistry, Philipps University Marburg, Marburg 35043, Germany; ‡ ISIS Pulsed Neutron and Muon Facility, 97008 STFC Rutherford Appleton Laboratory, Chilton OX11 0QX, U.K.

## Abstract

Phosgene (COCl_2_) is an important industrial reagent but its gaseous state and limited availability limit laboratory use. Diphosgene and triphosgene are safer surrogates, yet their solid-state structures and vibrational properties remain poorly documented. Here we report a comprehensive investigation of both compounds combining crystallography, calorimetry, spectroscopy, and quantum chemical calculations. A new polymorph of diphosgene (β-diphosgene) was discovered. Differential scanning calorimetry revealed rare cold crystallization behavior, i.e., crystallization of a supercooled melt only during subsequent heating. Solid-state DFT calculations reproduced lattice parameters and clarified the thermodynamic balance between the polymorphs. Infrared, Raman, and inelastic neutron scattering spectra of diphosgene and triphosgene were measured and fully assigned with the aid of periodic DFT calculations, providing the first complete solid-state vibrational characterization of these compounds.

## Graphical abstract

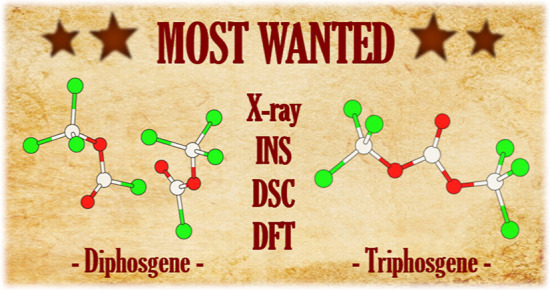

## Introduction

Since its discovery by Davy in 1812, phosgene (COCl_2_) has become one of the most important reagents in halogenation and acylation reactions, underpinning the large-scale industrial production of polyurethane precursors, pharmaceuticals, and agrochemicals.^1^ In stark contrast to its megaton-scale use in industry, phosgene is rarely encountered on the laboratory scale. Commercially, it is not available as a neat compound (in quantities lower than e.g. 40 tons), but only as solutions in toluene or hexane. In research laboratories, phosgene is typically substituted by thiophosgene (CSCl_2_), which exhibits somewhat similar reactivity, or by its formal di- and trimers, diphosgene and triphosgene (see Scheme 1). Their names are misleading, however, as they are not true oligomers of phosgene but rather act as sources of two or three equivalents.^1–4^ Compared to phosgene, their reactivity is significantly lower: phosgene reacts more than 2 orders of magnitude faster than triphosgene, as shown by methanolysis.^5^ Under typical reaction conditions with triphosgene, phosgene does not accumulate and remains below 10^–5^ M, enabling the safe usage of phosgene. When solvent-free phosgene is required, it can be generated in situ from di- or triphosgene by heating in the presence of catalysts such as charcoal or copper phthalocyanine at temperatures between 80–130 °C.^6–8^ Table 1 summarizes the physicochemical properties of phosgene, diphosgene and triphosgene.^1^

**Scheme 1:**
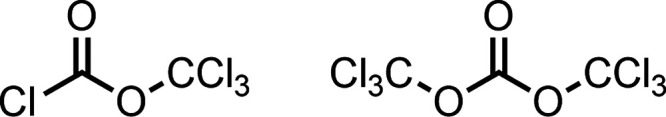
Representation of Diphosgene (Left) and Triphosgene (Right)

**Table 1:** Physicochemical Properties of Phosgene, Diphosgene and Triphosgene^c^

properties	phosgene	diphosgene	triphosgene
phase at 300 K	colorless gas	colorless liquid	colorless solid
molecular formula	COCl_2_	C_2_O_2_Cl_4_	C_3_O_3_Cl_6_
bp. [°C]	7.56	128	203–206
mp. [°C]	–127.8	–57	79–83
density [g cm^–3^]	1.38 (20 °C)	1.65 (14 °C)	1.629 (80 °C)
vapor pressure [bar]	1.09 (10 °C)	0.74–0.77 (20 °C)	0.12 (25 °C)
LC_50_ [mg m^3^]^a^	7.2	13.9	41.5
*k*_obs_ [s^–1^]^b^	1.7×10^–2^	9.1×10^–4^	1.0×10^–4^

a Inhalation for 240 min in a vapor atmosphere.^15^

b Pseudo-first-order rate constant for the reaction of 0.01 M with 0.3 M methanol in CDCl_3_ at 25 °C.^5^

c Data taken from ref 1.

Originally mentioned in 1887, the liquid diphosgene Cl­(CO)­OCl_3_ was developed as a less volatile phosgene alternative,^9^ but later gained notoriety due to its misuse during World War I.^6^ It can be synthesized conventionally by radical chlorination of methyl chloroformate under UV irradiation.^9^ Although its physicochemical properties have long been known, the first infrared spectroscopic data were published only in 1957 and revisited in 2006, including single-crystal X-ray analysis.

Triphosgene is a crystalline solid with a significant vapor pressure of ∼0.12 bar at ambient temperature.^1^ It was first prepared in 1880 by radical chlorination of dimethyl carbonate,^10^ and its chemical and physical properties were documented by Hentschel in the same decade.^9,11,12^ Its molecular structure, (Cl_3_CO)_2_CO, was proposed based on infrared (IR) spectroscopy and confirmed by X-ray diffraction in 1971.^13,14^ Notably, its chemistry remained largely unexplored until the late 1990s, when researchers started to use it as a phosgene substitute.^3^

Although the chemistry of diphosgene and triphosgene is well documented and both compounds have been structurally characterized, spectroscopic data remain scarce. Herein, we present a new polymorph of diphosgene and a revisited structure of triphosgene, providing fresh insight into their solid-state chemistry. We revisit the vibrational spectroscopy of both di- and triphosgene using inelastic neutron scattering (INS) as a complementary technique to Raman and IR methodology.

## Experimental Section

All manipulations were carried out in a ventilated fume hood. Diphosgene (Acros Organics, 99%) and triphosgene (abcr chemicals, 98%) were purchased commercially and used as received.

### X-Ray Diffraction

Single-crystal X-ray diffraction data were collected using a StadiVari (Stoe, Darmstadt) diffraction system equipped with a mirror monochromated Cu Kα radiation (λ = 1.54186 Å, Xenocs Microfocus Source) and a Pilatus 300 K detector. Triphosgene crystals were selected under Paratone-N oil, mounted on micromount loops and quench-cooled using an Oxford Cryosystems open flow N_2_ cooling device. Unless otherwise stated, data were collected at 100 K and processed using the X-Area software suite, which included unit cell parameter refinement and interframe scaling (which was carried out using LANA within X-Area). Structures were subsequently solved using direct methods (SHELXT)^16^ and refined on *F* ^2^ with SHELXL^17^ using the Olex2 ^18^ user interface. Crystal structure illustrations were generated using DIAMOND software.^19^ For further details regarding single-crystal refinements see Supporting Information.

### Growth of Single Crystals of Triphosgene

Single crystals of triphosgene were grown by sublimation at ambient pressure at 0 °C in the storage bottle in the fridge.

### Growth of Single Crystals of β-Diphosgene

Diphosgene was filled into a glass capillary (diameter 0.5 mm, Hilgenberg) and flame-sealed at ambient pressure. The capillary was mounted on the goniometer at room temperature. A polycrystalline sample was obtained by cooling to 100 K, followed by heating to ∼160 K at a rate of 180 K min^–1^. The sample was then incrementally heated in 1 K steps until partial melting was observed at 211 K. A suitable single crystal was subsequently grown through Ostwald ripening, facilitated by a sinusoidal temperature profile oscillating around the melting point ∼216 K.

### Hirshfeld Surface Analysis

Hirshfeld surface analyses were conducted with the Crystal Explorer (version 21.5) software suite.^20,21^

### Differential Scanning Calorimetry

Differential scanning calorimetry measurements were performed on a heat flow differential scanning calorimeter model STARe System DSC 3 (Mettler Toledo, Columbus, Ohio, United States). A constant stream of nitrogen (10 cm^3^ min^–1^) was used as purging gas. Diphosgene (16.3 mg, 0.082 mmol) was placed in a 40 μL aluminum crucible with a pin profile which was subsequently closed with a press. The data was evaluated using the STARe program (Mettler Toledo). The extrapolated onset melting-temperature was defined by the intersection point of the extrapolated baseline and the inflectional tangent at the beginning of the melting peak. The corresponding melting enthalpy was determined by the absolute integral and the weighted sample (J g^–1^) of the heat flow signal and converted into kJ mol ^–1^.

### Vibrational Spectroscopy

Raman spectra were recorded on a Monovista CRS + confocal Raman microscope (Spectroscopy & Imaging GmbH) using a 532 nm solid-state laser and a 1800 grooves/mm grating. Samples were flame-sealed in borosilicate capillaries at room temperature and measured at room temperature, 193 and 350 K (Linkam temperature-controlled stage). The accumulation was 5 × 5 s.

Attenuated total internal reflection (ATR) infrared spectra were recorded at room temperature using a Bruker Alpha II FTIR spectrometer (64 scans at 4 cm^–1^ resolution).

INS spectra were recorded using the high resolution, broad band spectrometer TOSCA^22,23^ at the ISIS Neutron and Muon Facility.^24^ The samples (16.1 g diphosgene and 7.9 g triphosgene) were each loaded into In-wire sealed aluminum cells, cooled to ∼10 K in a closed cycle refrigerator and measured for ∼12 h. The as-recorded time-of-flight data were converted to energy transfer using Mantid v6.10.0.^25^

### Quantum Chemical Calculations

Solid-state calculations on the energetics and thermodynamics were carried out with the CRYSTAL17 program package.^26^ PBE0 hybrid density functional method (PBE exchange–correlation and 25% exact HF exchange) with Grimme’s D3 dispersion correction was used (D3 with Becke-Johnson damping and three-body ABC correction).^27,28^ Molecular def2-TZVP polarized triple-ζ-valence basis were applied for all atoms without any modifications.^29^ Reciprocal space was sampled by Monkhorst–Pack type k-meshes for triphosgene: 3 × 3 × 2, β-diphosgene at 100 K: 3 × 3 × 2 and literature known α-diphosgene: 6 × 2 × 4.^30^ For the evaluation of the Coulomb and exchange integrals (TOLINTEG), tolerance factors of 8, 8, 8, 8, and 16 were used for all calculations. Atomic positions and lattice parameters were fully optimized within the constraints of space group symmetry. Default DFT integration grids and optimization convergence thresholds were applied in all calculations. Harmonic frequency calculations were carried out with the finite-displacement approach implemented in CRYSTAL.^31^ Harmonic frequency calculations at the Γ point showed all studied structures to be true local minima with no imaginary frequencies. Gibbs free energies were obtained for α-diphosgene using a 2 × 1 × 1 phonon supercell that has the same number of atoms as β-diphosgene in its primitive cell.

For calculations of the INS spectra, the plane wave code CASTEP^32^ was used with on-the-fly generated norm-conserving pseudopotentials, the PBE functional^27^ and the Tkatchenko-Scheffler dispersion correction.^33^ The plane-wave cutoff was 1020 eV and the Brillouin zone sampling of electronic states used a 8 × 5 × 6 Monkhorst–Pack grid (72 *k*-points) for diphosgene and a 6 × 7 × 6 Monkhorst–Pack grid (72 *k*-points) for triphosgene. In both cases, the initial structure was that determined at 100 K in the present work. Phonon transition energies were obtained by diagonalization of dynamical matrices computed using density-functional perturbation theory.^34^ Phonon dispersion was calculated along high symmetry directions throughout the Brillouin zone. Dynamical matrices were computed on a regular grid of wavevectors throughout the Brillouin zone and Fourier interpolation was used to extend the computed grid to the desired fine set of points along the high-symmetry paths.^35^ The calculated INS spectrum was generated from the atomic displacements in each mode that are part of the CASTEP output, using AbINS.^36^

## Results and Discussion

### Crystal Structure of β-Diphosgene

Our investigations began with a redetermination of the crystal structure of diphosgene. Previously, its structure was reported from a crystal grown in a cooling stream under heating with a CO_2_ laser.^37^ We sought to determine whether a crystal obtained by simple cooling would exhibit the same structure. A borosilicate glass capillary (0.3 mm outer diameter) was filled with a small amount of diphosgene and flame-sealed at ambient pressure. The capillary was mounted on the goniometer of the diffractometer and cooled using an open-flow cryostat. Cooling below the reported melting temperature of 216.15 K^1^ (see Table 1) did not yield crystals and, in fact, produced no solid material at all, as confirmed both by test measurements and visual inspection with the attached camera. Only upon subsequent heating from 100 K to ∼160 K, a polycrystalline sample formed, indicating a “cold crystallization” process (discussed further below). The polycrystalline sample was then incrementally heated in 1 K steps until partial melting was observed at 211 K. A suitable single crystal was subsequently grown through Ostwald ripening. Test measurements confirmed the presence of several single crystals. Full data sets were collected at 200 and 100 K, and the reflections of the strongest diffracting crystal were integrated.

With this strategy, crystals of a new diphosgene modification were grown, which we designate as β-diphosgene; the previously reported polymorph will be referred to as α-diphosgene. β-Diphosgene crystallizes in the monoclinic crystal system with space group *P*2_1_/*n* (No. 14). At 200 K, the unit cell parameters are *a* = 11.7485(5) Å, *b* = 7.4109(2) Å, *c* = 15.4942(6) Å, and β = 94.581(3)°. At 100 K, the parameters refine to *a* = 11.6703(3) Å, *b* = 7.3870(2) Å, *c* = 15.3222(4) Å, and β = 94.574(2)°. Lowering the temperature for the second measurement did not induce a phase transition. The unit cell contains eight molecules (*Z* = 8), comprising two crystallographically independent molecules in the asymmetric unit. Each adopts the *syn* conformation, defined by the orientation between the C=O and O–CCl_3_ bonds, as illustrated in Figure 1. All atoms occupy Wyckoff position 4*e* (site symmetry 1).

**Figure 1:**
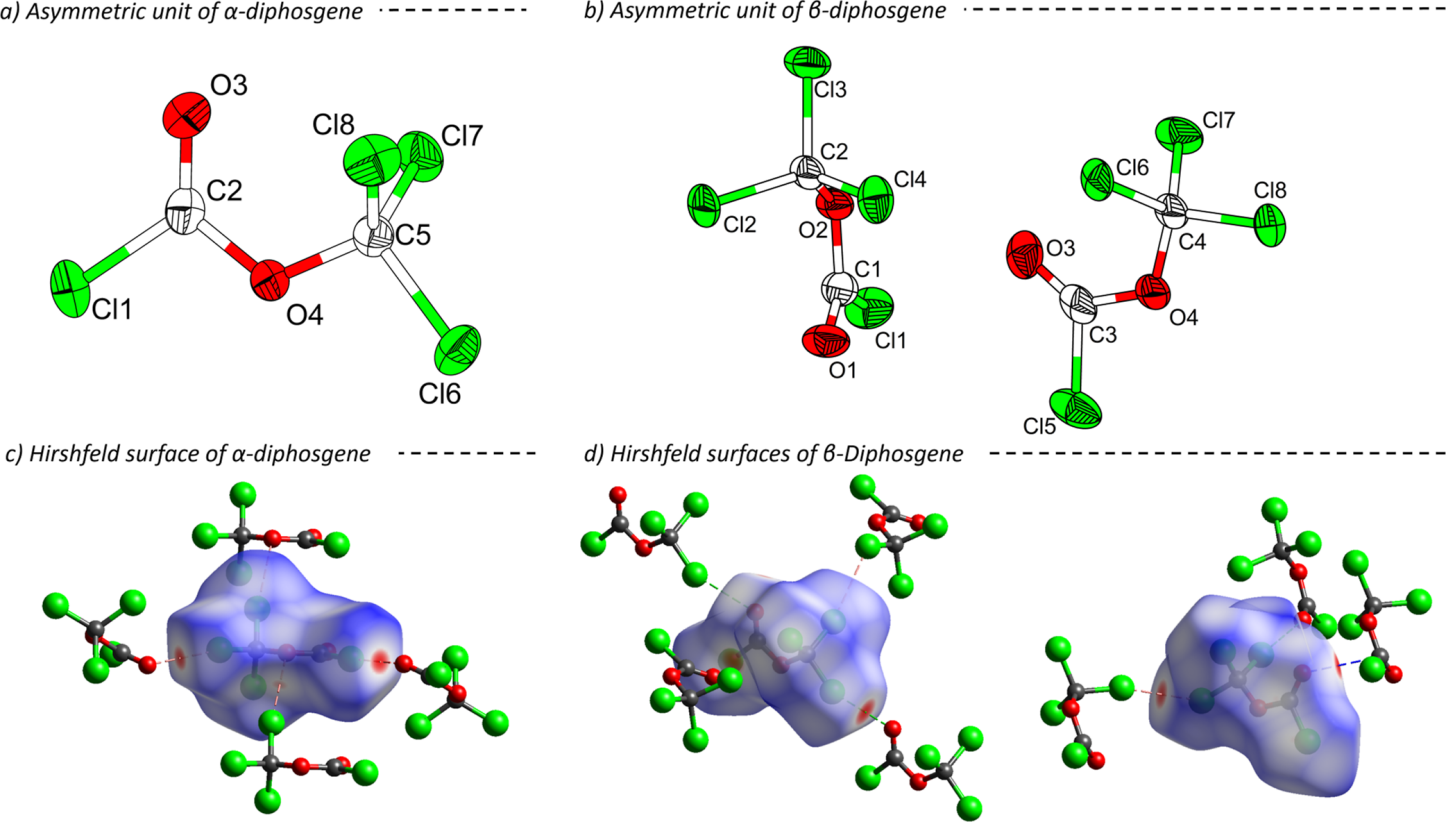
Asymmetric units of the crystal structures of α-diphosgene (a, measured at 150 K)^37^ and β-diphosgene (b, measured at 100 K) with ellipsoids drawn at 60% probability level. Hirshfeld surfaces of α-diphosgene (c) and both independent molecules of β-diphosgene (d). Atoms are drawn with arbitrary radii. Short contacts are marked with dashed lines.

### Comparison of the Crystal Structures of α- and β-Diphosgene

Both polymorphs of diphosgene crystallize in space group *P*2_1_/*n* (No. 14). Their crystal structures, however, are not isotypical. The unit cell of β-diphosgene is twice the size of that of α-diphosgene, comprising eight instead of four molecules (see Table 2). β-Diphosgene contains two crystallographically independent molecules in the asymmetric unit, whereas α-diphosgene contains only one. All molecules adopt the *syn* conformation. It has been shown that the *anti* conformer is energetically disfavored and is also not observed in gas-phase vibrational spectra.^37^ There is no evidence that the two polymorphs are linked by any direct group–subgroup relationship. Careful examination during data processing and refinement did not indicate overlooked symmetry, as checked with PLATON (ADDSYM).^38^ Lastly, both polymorphs differ in their respective packing motifs.

**Table 2:** Unit Cell Parameters of α-Diphosgene^37^ and β-Diphosgene Determined by X-Ray Diffraction

	α-diphosgene	β-diphosgene	
	150 K	100 K	200 K
*a* [Å]	5.5578(5)	11.6703(3)	11.7485(5)
*b* [Å]	14.2895(12)	7.3870(2)	7.4109(2)
*c* [Å]	8.6246(7)	15.3222(4)	15.4942(6)
β [°]	102.443(2)	94.574(2)	94.581(3)
*V* [Å^3^]	668.86(10)	1316.70(6)	1344.72(9)
*T* [K]	150	100	200
*Z*	4	8	8

The crystal packing of both polymorphs is generally dominated by Cl···O short contacts, which are particularly pronounced in α-diphosgene, as illustrated by the Hirshfeld surface in Figure 1. The corresponding 2D fingerprint plots of the intermolecular contacts are shown in the Supporting Information. The Cl···O distances in α- and β-diphosgene are 3.1246(8) and 3.184(4) Å, respectively, both shorter than the sum of the van der Waals radii (3.27 Å), indicating strong attractive intermolecular forces.^39^ In the fingerprint plots, these interactions correspond to the characteristic “spikes” at *d*_e_/*d*_i_ values of 1.4/1.6 Å and 1.6/1.4 Å in β-diphosgene. Additionally, Cl···Cl interactions with a length of 3.3000(14) Å are present in β-diphosgene between a pair of crystallographically independent molecules. The packing of the new modification is further stabilized by short dipole–dipole interactions, *d*(C···O) = 3.020(5) Å, between the oxygen and carbon atoms of adjacent carbonyl groups.

The arrangement of molecules within the unit cell also differs between α- and β-diphosgene. In the latter, the packing is dominated by “pairs” of crystallographically equivalent molecules (see Figure 2), showing strong intermolecular C=O···Cl contacts, weaker Cl···O­(alkoxy) interactions, and Cl···Cl contacts to neighboring molecules of the second asymmetric unit component. In β-diphosgene, one molecule of the asymmetric unit is aligned almost parallel to the *a*,*b* plane, while the other is oriented orthogonally within the *b*,*c* plane of the unit cell. By contrast, in α-diphosgene the molecules are slightly tilted and not perfectly aligned in a plane, resulting in a less tidy-looking packing motif. In this polymorph, each molecule is surrounded by six neighbors via Cl···O short contacts.

**Figure 2:**
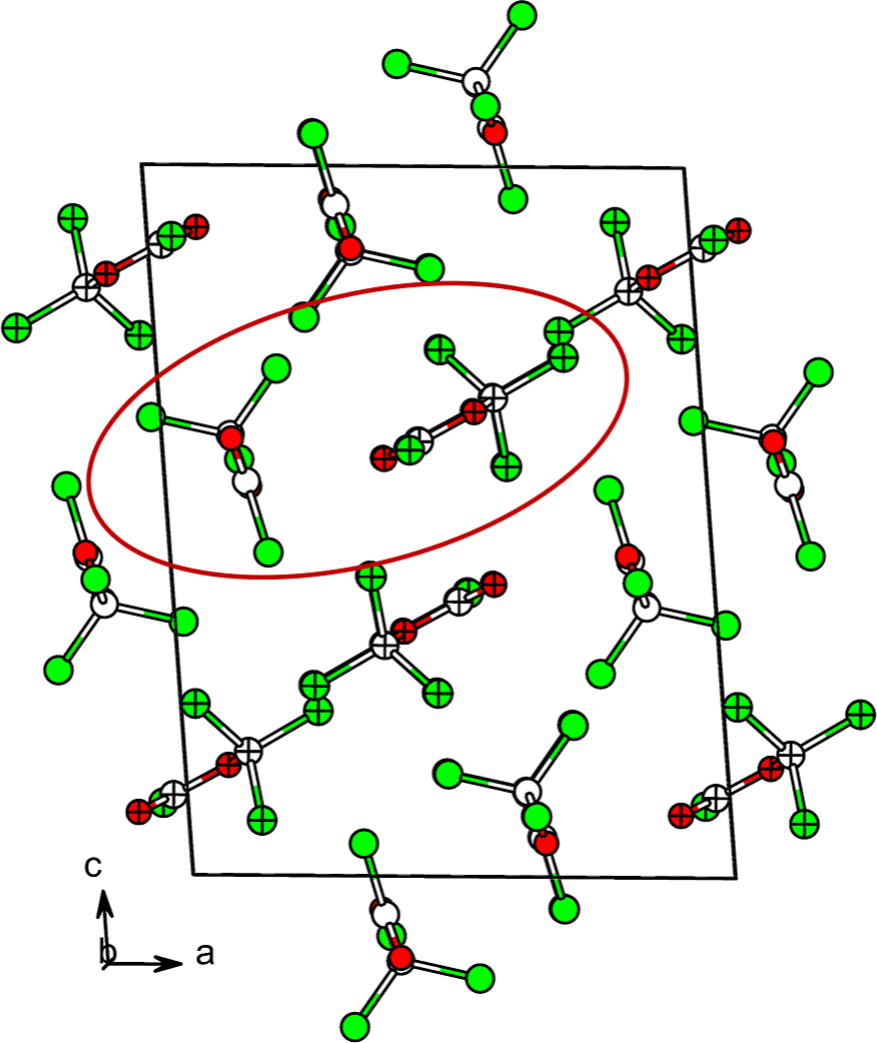
Arrangement of molecule “pairs” in the crystal structure of β-diphosgene. The asymmetric unit is shown in the circle with the two crystallographically independent molecules with different patterns.

### Redetermination of the Crystal Structure of Triphosgene

Even at 0 °C, triphosgene exhibits substantial vapor pressure, leading to sublimation.^1^ Consequently, single crystals of triphosgene were selected directly from the storage bottle kept in a refrigerator. At 100 K, triphosgene crystallizes in the monoclinic crystal system with space group *P*2_1_/*c* (No. 14). The refined unit cell parameters are *a* = 9.7241(7) Å, *b* = 8.7991(7) Å, *c* = 11.1583(9) Å, and β = 91.330(6)°, with four formula units in the unit cell (*Z* = 4) and one independent molecule in the asymmetric unit (see Figure 3). All atoms occupy Wyckoff position 4*e* (site symmetry 1). This model closely mirrors the previously reported crystal structure. Although the literature does not specify the measurement temperature, the reported lattice parameters (*a* = 9.814(8) Å, *b* = 8.879(4) Å, *c* = 11.245(4) Å, β = 91.7(1)°) suggest that the earlier study was conducted at room temperature.^14^ In the crystal structure, triphosgene adopts a planar geometry of the central moiety in which the two–OCl_3_ groups are oriented *syn* with respect to the carbonyl group. Individual molecules are linked by weak O···Cl short contacts.

**Figure 3:**
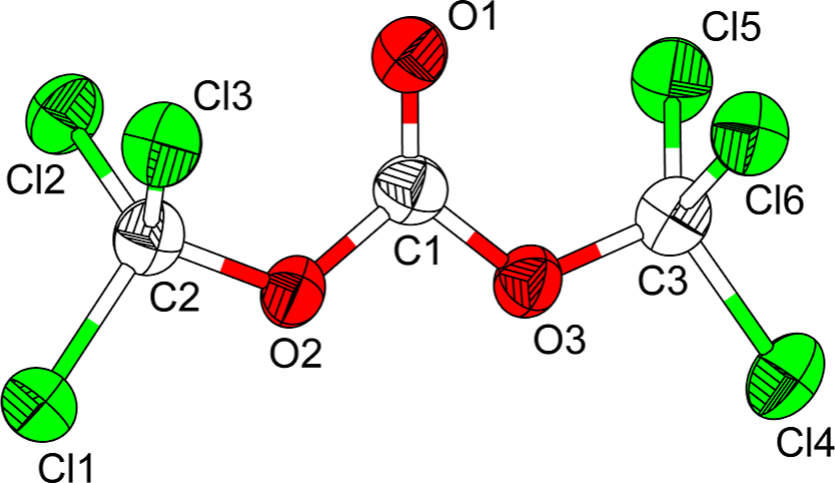
Molecular structure of one independent triphosgene molecule in the solid state measured at 100 K. Atoms are drawn with 60% displacement ellipsoids.

### Molecular Structure Comparison Both of α- and β-Diphosgene, and Triphosgene

In the crystal structure of β-diphosgene, both crystallographically independent molecules exhibit similar geometrical parameters within the 3σ range. In triphosgene, the two chemically identical halves of the molecule likewise show comparable interatomic distances within the 3σ range. A comparison of the structural parameters among α- and β-diphosgene,^37^ triphosgene, phosgene,^40^ dimethyl carbonate,^41^ methyl chloroformate,^42^ and trifluoromethyl chloroformate^43^ is provided in Table 3.

**Table 3:** Comparison of Selected Interatomic Distances of Di- and Triphosgene at 100 K and Chosen Compounds for Comparison^d^

	α-diphosgene^c^ ^37^	β-diphosgene (1)	β-diphosgene (2)	triphosgene	phosgene^40^	dimethyl carbonate^b^ ^,^ ^41^	methyl chloroformate^42^	trifluoromethyl chloroformate^43^
C=O	1.1802(12)	1.172(5)	1.167(5)	1.188(5)	1.184(2)	1.219(2)	1.195(2)	1.164(4)
(CO)–O	1.365(1)	1.356(5)	1.362(5)	1.361(6)^a^	–	1.337(2)	1.309(2)	1.367(4)
CX_3_–O	1.419(1)	1.418(5)	1.407(5)	1.414(5)^a^	–	1.456(2)	1.462(2)	1.386(3)
(CO)–Cl	1.729(1)	1.732(4)	1.735(4)	–	1.725(2)^a^	–	1.7502(13)	1.716(3)
C–X	1.7627(9)^a^	1.759(4)^a^	1.759(4)^a^	1.762(5)^a^	–	–	–	1.310(4)
∠O=C–O	127.86(9)	127.8(4)	128.1(4)	128.7(4)^a^	–	125.58(11)	128.78(11)	126.9(3)
∠X–C=O	124.80(7)	125.2(3)	124.8(3)	–	123.9(2)	–	122.47(10)	125.3(2)
∠X–C–O	107.34(6)	107.0(3)	107.0(3)	–	–	–	108.75(9)	107.8(2)

a Mean values.

b Neutron powder data at 82 K.

c SCXRD at 150 K.

d Parameters in [Å] and [°].

C=O carbonyl bond lengths of both diphosgene modifications and triphosgene lie between 1.167(5) and 1.188(5) Å, shorter than the additive covalent radii (1.24 Å).^44^ These values are comparable to those in trifluoromethyl chloroformate and phosgene, about 0.03 Å shorter than in methyl chloroformate, and even shorter than in dimethyl carbonate (1.219(2) Å).

For the (CO)–Cl bond, similar distances are observed in both diphosgene modifications, ranging between 1.729(1) and 1.735(4) Å. These values are close to that in phosgene (1.725(2) Å) and slightly shorter than in methyl chloroformate (1.7498(13) Å). The difference likely arises from the slightly lower group electronegativity of the O–CCl_3_ substituent compared to Cl in phosgene.^45^ In contrast, and similar to methyl chloroformate, the C=O bond length in dimethyl carbonate (1.219(2) Å) is longer due to the lower group electronegativity of O–CH_3_, compared to halogen-containing substituents.

The (CO)–O and O–C*X*_3_ bond lengths in all modifications of di/triphosgene are very similar, lying between 1.356(5) and 1.365(1) Å, and 1.407(5) and 1.419(1) Å, respectively. In trifluoromethyl chloroformate, the corresponding bond lengths are 1.367(4) and 1.386(3) Å, reflecting the higher group electronegativity of O–CF_3_. Conversely, substitution of halogen atoms by hydrogen, as in dimethyl carbonate and methyl chloroformate, lowers the group electronegativity and results in longer bond lengths: (CO)–O = 1.337(2) Å and O–CH_3_ = 1.456(2) Å in dimethyl carbonate. The O–C*X*_3_ bond length is more strongly affected by group electronegativity than the (CO)–O bond.

In diphosgene, the molecular structure of the central motif approaches planarity with a torsion angle of ≈178.0(5)° for ∠(Cl–C–O–C), while the–CCl_3_ group adopts a staggered position relative to the carbonyl group. Similar to methyl chloroformate, the –CCl_3_ group is oriented *syn* with respect to the C=O bond. In triphosgene, both trichloromethane groups are slightly tilted in a less pronounced eclipsed fashion relative to each other, resulting in nearly *C*_2v_ symmetry with a torsion angle of ∠(C–O–C–C) = 176.5(4)°. Again, both –CCl_3_ groups are oriented *syn* to the C=O bond. The ∠(O=C–O) angle in di- and triphosgene lies between 127.8(4)° and 128.7(4)°, similar to methyl chloroformate but about 0.9° and 3.1° smaller than in trifluoromethyl chloroformate and dimethyl carbonate, respectively.

### Differential Scanning Calorimetry of Diphosgene

During single-crystal growth of diphosgene in the capillary, no solidification was observed down to 80 K. While the formation of supercooled melts is not uncommon, the extraordinary range in this case warranted further investigation. Upon reheating the sample, rapid solidification was observed above ∼160 K. To determine whether this behavior could be reproduced outside of a capillary, to extract melting and solidification points, and to identify possible phase transitions, we performed low-temperature differential scanning calorimetry (DSC). In contrast, the DSC data of triphosgene has already been published and does not indicate the formation of different polymorphs.^3^

Diphosgene (16.30 mg, 0.082 mmol) was placed in a 40 μL aluminum crucible with a pin profile, which was subsequently sealed with a press. Initial measurements were carried out at rates of 10 K min^–1^ and 4 K min^–1^, ranging from ambient temperature to 123 K (see Supporting Information). No significant differences were observed between the corresponding spectra. Subsequently, three measurements were performed in the range between 123 and 233 K using a cooling and heating rate of 4 K min^–1^. During cooling, diphosgene remained in a supercooled state without crystallization down to 123 K. Reheating resulted in solidification at ∼170 K, which is 46 K below the reported melting point (Figure 4; the three separate curves are provided in the Supporting Information).^1^ The DSC profiles for all three cycles nearly overlapped; however, solidification during the third cycle was not observed. Correspondingly, the melting signal was absent, underlining the strong tendency of diphosgene to form supercooled liquids.

**Figure 4:**
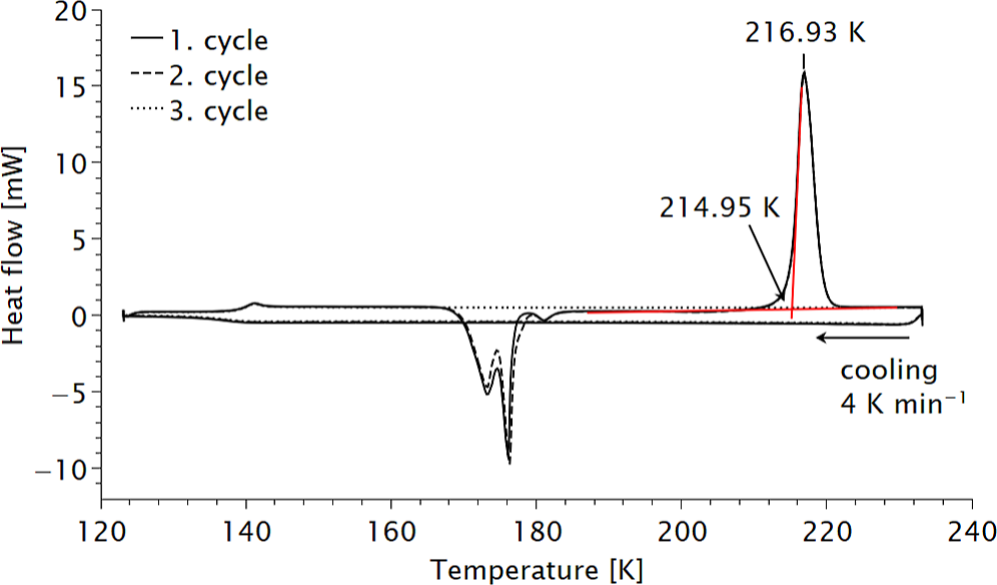
Low-temperature differential scanning calorimetry data of diphosgene. Three consecutive measurements were performed. The curves for solidification and melting temperature coincide. For details of the fit see Supporting Information.

The first major thermal effect, observed at 140 K, corresponds to the endothermic glass transition (*T*_g_) of the amorphous phase, marking the transition from a rigid to a rubbery state due to molecular relaxation and the release of molecular mobility. Upon further heating above *T*_g_, the amorphous state gains mobility, and an exothermic double peak associated with cold crystallization is observed at *T*_c_ = 170 K. This event may be accompanied by a double crystallization peak.^46^ Following crystallization, another exothermic anomaly is observed, which is absent in the amorphous phase without prior solidification. Finally, melting of the sample occurs at *T*_m_ = 215 K. The same DSC curve was reproduced in a second measurement, confirming all thermal events.

This unique phenomenon, known as cold crystallization, in which crystallization first occurs upon heating below the melting point, is very rare for small organic molecules. It is typically observed in polymers, ionic liquids, and metal complexes.

### Quantum Chemical Calculations

In order to gain a deeper insight into the formation of the two different modifications of diphosgene depending on the crystallization method, we performed solid-state quantum chemical calculations using the CRYSTAL17 software package.^26^ We employed the dispersion-corrected hybrid density functional theory (DFT) method at the PBE0-D3­(BJ-ABC)/def2-TZVP level of theory (see computational details) to determine the fully optimized geometries of both α- and β-diphosgene in their crystal structures at 0 K. Overall, the lattice parameters are well reproduced, with only small deviations compared to the experimentally determined crystal structure for α-diphosgene at 150 K: *a* = 5.571 Å (+0.2%), *b* = 14.265 Å (−0.2%), *c* = 8.572 Å (−0.6%) and β = 102.79° (+0.3%) and β-diphosgene at 100 K: *a* = 11.732 Å (+0.5%), *b* = 7.392 Å (+0.1%), *c* = 15.341 Å (+0.1%) and β = 95.01° (+0.5%). Additionally, one also can compare the corresponding lattice energies at 0 K, defined as the energy difference between the crystalline solid per formula unit and one individual molecule in the gas phase. The lattice energies of α- and β-diphosgene are 59.9 and 59.6 kJ mol^–1^, respectively.

To study the thermodynamics of the two polymorphs of diphosgene in the solid state, we compared the Gibbs free energy and the entropy at various temperatures below the melting temperature in the range 200 to 50 K (see Figure 5). For this purpose, frequencies and thermodynamic properties were calculated within the harmonic approximation as implemented in CRYSTAL17 using a 2 × 1 × 1 phonon supercell for α-diphosgene to match the number of atoms in the unit cell of β-diphosgene. At 0 K, α-diphosgene is slightly favored by 0.3 kJ mol^–1^ per formula unit compared to β-diphosgene.

**Figure 5:**
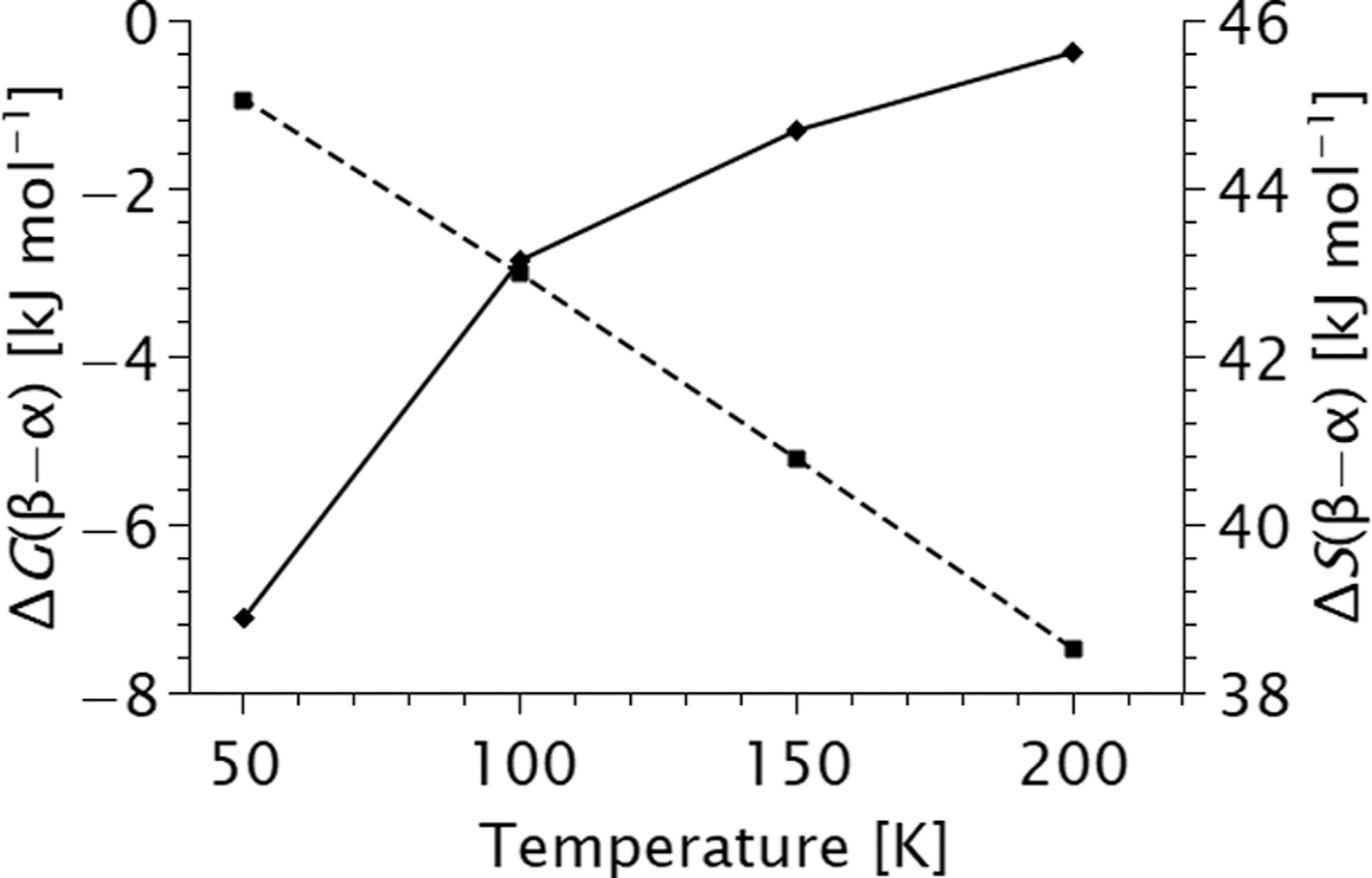
Difference in entropy (—) and Gibbs free energy (---) between β- and α-diphosgene at different temperatures. Level of theory: DFT-PBE0-D3­(BJ-ABC)/def2-TZVP.

In contrast, the Gibbs free energy reveals that β-diphosgene is thermodynamically favored between 50 and 200 K. Just below the melting temperature, at 200 K, the Gibbs free energy is 7.5 kJ mol^–1^ lower than that of α-diphosgene, with the difference decreasing to 0.9 kJ mol^–1^ at 50 K. At the same time, the entropy is larger for β-diphosgene throughout the entire temperature range by about ∼42 kJ mol^–1^. Based on the data, we assigned β-diphosgene as the more stable polymorph, while α-diphosgene is higher in energy at every temperature.

### Vibrational Spectroscopy of Diphosgene and Triphosgene

Reports of the vibrational spectroscopy of diphosgene and triphosgene are scarce. A 1957 paper^13^ reported the strongest infrared bands of the materials. At the time, it was debated whether diphosgene and triphosgene were dioxacyclobutane and dioxan type structures respectively or open-chain. From the presence of the carbonyl absorption around 1800 cm^–1^, the author deduced that the correct structures must be open-chain. The only other report we are aware of is the 2006 structural study of diphosgene^37^ which also included an analysis of the gas and liquid phase spectra. There are no reports of solid state spectra for either material.

Figures 6 and 7 show the infrared, Raman and inelastic neutron scattering (INS) spectra of diphosgene and triphosgene, respectively. The complementarity of the three forms of spectroscopy is evident. Infrared spectroscopy is highly sensitive to the polar modes, largely those involving C and O, however, strong electrical anharmonicity results in very broad modes. Raman spectroscopy provides a wider energy range and much sharper modes. INS^47^ is a form of spectroscopy that does not depend on the interaction of photons with electrons and as a consequence there are no selection rules. The INS intensity is proportional to the amplitude of vibration and the neutron scattering cross section. In the harmonic approximation, the amplitude of vibration is inversely dependent on the wavenumber, thus low energy modes are favored. The cross section is both element and isotope dependent and ^35^Cl (76% abundance) has a total scattering cross section of 21.8 barn (1 barn = 10^–28^ m^2^) *c*.*f*. C = 5.6 and O = 4.2 barn, respectively. Thus, modes involving Cl will dominate the spectrum, which explains the weakness of the carbonyl stretch at ∼1800 cm^–1^ in the INS spectra.

**Figure 6:**
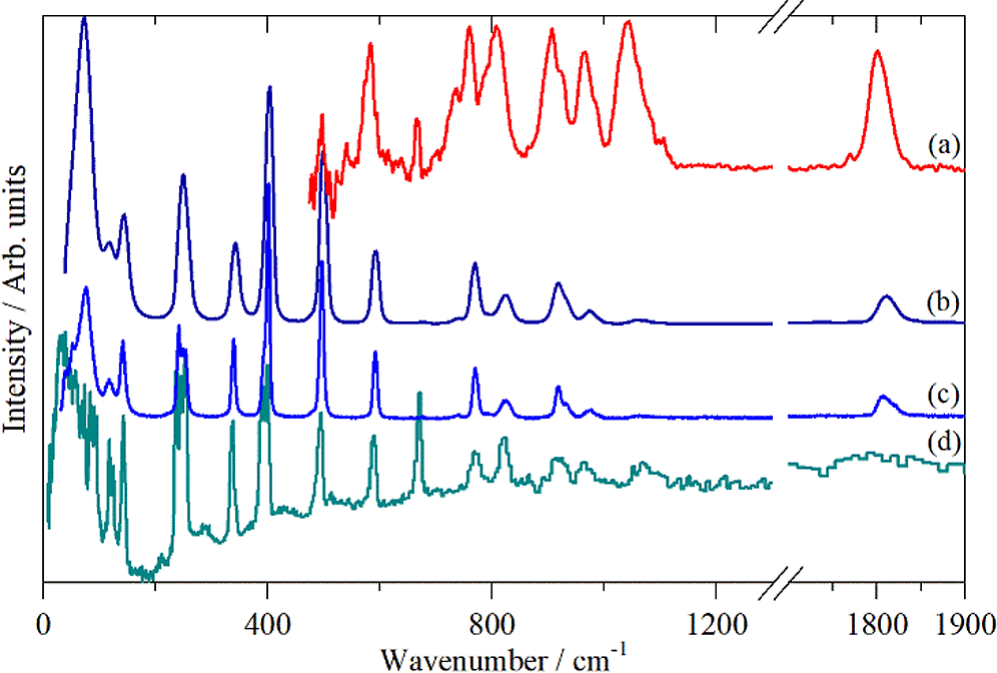
Vibrational spectra of diphosgene: (a) infrared (liquid at room temperature), (b) Raman (liquid at room temperature), (c) Raman (solid, 193 K) and (d) INS (solid at 15 K).

**Figure 7:**
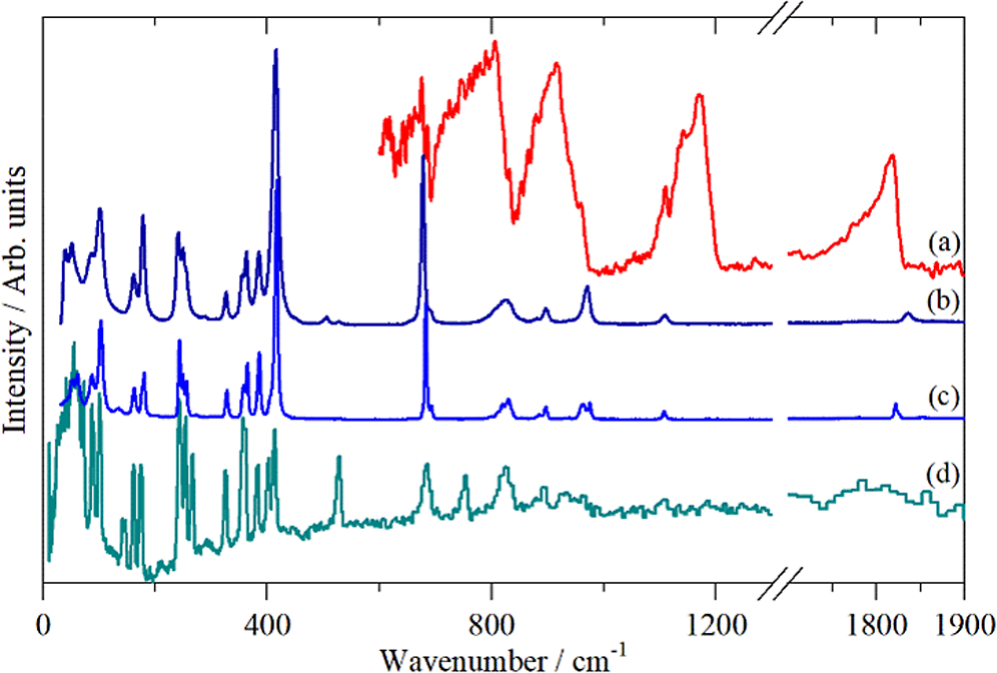
Vibrational spectra of triphosgene in the solid state: (a) infrared (room temperature), (b) Raman (liquid at 350 K), (c) Raman (solid at room temperature) and (d) INS (15 K).

#### Diphosgene

This has been studied in the gas phase, the liquid state and in a low-temperature matrix.^37^ The authors concluded that the *syn* conformer (where the CCl_3_ group is “*cis*” to the carbonyl) was significantly more stable than the *anti* conformer (where the CCl_3_ group is “*trans*” to the carbonyl). Their spectra support this conclusion and the *syn* conformer is exclusively found in the solid state. Our structural and spectroscopic studies are in complete agreement with this finding. In particular, there is very little difference between the liquid and solid-state Raman spectra (Figure 6b,c) of diphosgene.

The highest possible symmetry of the free (i.e., gas phase) molecule is *C*_S_. α- and β-diphosgene both crystallize in the monoclinic space group *P*2_1_/*n* (No. 14) with four and eight formula units in the primitive cell (*Z* = 4, 8) respectively, all on general sites (*C*_1_ symmetry). This means that each mode of the isolated molecule gives four/eight modes in the solid-state. As seen from the correlation Tables S5 and S6, half of these are infrared allowed (the ungerade modes) and the other half are Raman allowed (the gerade modes). Under our measurement conditions, α-diphosgene is obtained. Consequently, each infrared and Raman vibrational mode is expected to appear as a doublet, while each INS mode should split into a quadruplet (all the modes contribute to the INS spectrum as there are no selection rules). However, the spectra show no evidence for factor group splitting. The multiplets observed at 250 and 395 cm^–1^ correspond to overlapping fundamental modes, as explained here in after. To enable a complete assignment of the spectra, periodic density functional theory (DFT) calculations of the complete cell of α-diphosgene is have been carried out. The resulting dispersion (variation of transition energy with wavevector) curves are shown in Figure S24. It can be seen, that apart from the acoustic translational modes, all of the modes are essentially flat across the entire Brillouin zone. In the absence of resolution or sample (e.g., poorly crystalline) broadening, the width of an INS peak is determined by the projection of the dispersion curves onto the energy axis. In the 300–800 cm^–1^ region the resolution of TOSCA is ∼10 cm^–1^,^48^ the bands in this region due to single modes have a width of ∼12 cm^–1^ i.e. nearly resolution limited, consistent with almost no dispersion being present.

Table S7 lists the calculated modes at Brillouin zone Γ point. It can be seen that for most of the internal modes, the factor group splitting is less than 10 cm^–1^, consistent with the INS spectrum.

#### Triphosgene

The only previous work we are aware of is the 1957 study by Hales et al.^13^ This only listed the strongest infrared peaks. The highest possible symmetry of the free (i.e., gas phase) molecule is *C*_2v_. Triphosgene crystallizes in the monoclinic space group *P*2_1_/*c* (No. 14) with four formula units in the primitive cell (*Z* = 4), all on general sites (*C*_1_ symmetry). The predictions from the correlation method (Table S8) are the same as for α-diphosgene, the only difference is that there are more modes because of the larger number of atoms in the molecule.

As with α-diphosgene, the periodic DFT calculations show essentially flat dispersion curves (Figure S25), consistent with the nearly resolution limited widths found in the INS spectrum, Figure 7d. Table S9 lists the calculated modes at the Brillouin zone Γ point, again for most of the internal modes, the factor group splitting is less than 10 cm^–1^.

### Assignment of the Spectra of α-Diphosgene and Triphosgene

Figure 8 shows the observed INS spectra and those calculated for α-diphosgene and triphosgene. It can be seen that there is reasonable agreement, enabling a complete assignment of the spectra. In practice, even with mode visualizations, this proved surprisingly difficult. There are two reasons for this. First, in a hydrogenous molecule, the modes are spread across 3000 cm^–1^ or so, whereas for these perchloro compounds it is only 1200 cm^–1^, (excluding the carbonyl stretch). Thus, the modes are much more congested. Second, in hydrogenous systems, mode descriptions are typically C–H stretch, C–H bend etc..: it is the hydrogen atom(s) that move and the carbon atom is largely stationary. For chloro compounds, this is not the case and the Cl atoms are stationary and the carbon atom(s) move. Hence, there is a perception bias to overcome in order to describe the modes.

**Figure 8:**
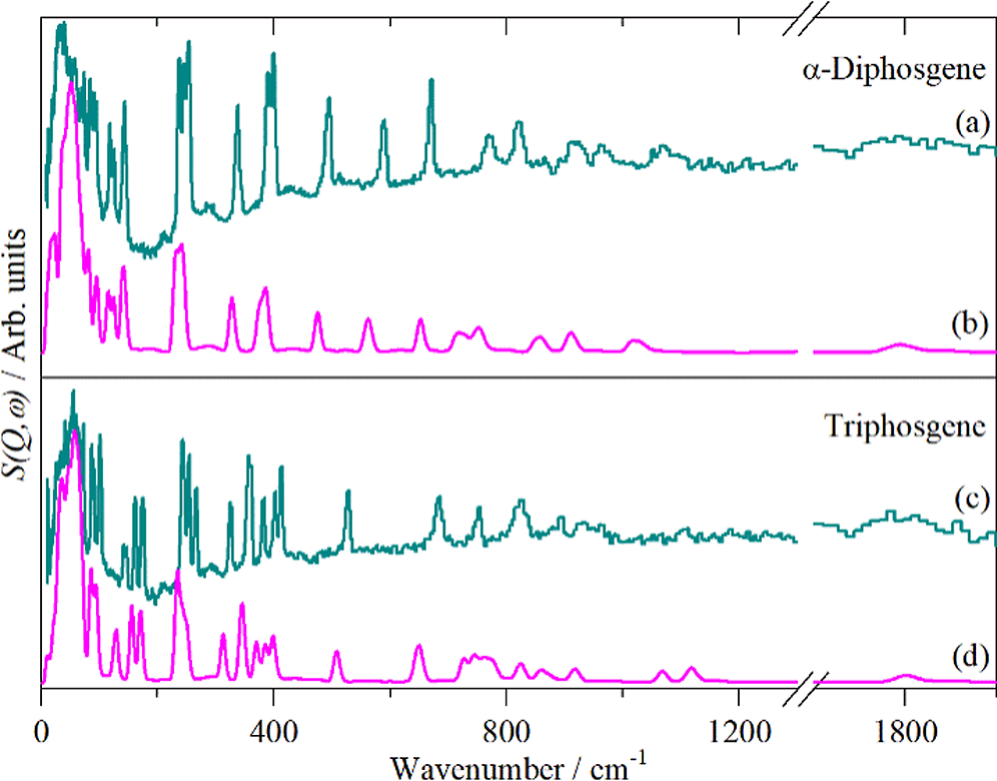
Comparison of measured ((a) and (c)) and calculated ((b) and (d)) INS spectra of α-diphosgene and triphosgene.

As the primitive cell’s of the two molecules both contain four molecules, to simplify the assignment process, the spectra of isolated (i.e., pseudo gas phase), idealized *C*_s_ (diphosgene) and *C*_2v_ (triphosgene) systems were also calculated, these are given in Tables S10 and S11, respectively. Full periodic calculations of the primitive cell across the entire Brillouin zone were then made. Table 4 lists all of the observed modes of α-diphosgene and triphosgene and their assignments. These are based on the calculated spectra and assignments of the complete primitive cell at the Brillouin zone Γ-point are given in Tables S7 and S9.

**Table 4:** Observed Infrared, Raman and INS Modes [cm^–1^] of α-Diphosgene and Triphosgene and Their Assignments

α-diphosgene				triphosgene			
infrared^a^	Raman	INS	assignment	infrared	Raman	INS	assignment
		69 m, 74 w					
		84 s				42 m	Libration
		91 m, 95 m				56 s,vbr	Libration
						74 s	CCl3 rock
					85 m	89 s	CCl3 rock
		117 m/126 m			101 s	103 s	CCl3 rock
						143 m/147 m	O(2)C(2) + C(2)O(3) ip torsion
	141 m	144 s	CCl3 rock		161 w	162 s	CCl3 rock
					177 m	175 s	O(1)C(2)O(3) bend
	240 m	238 s	Cl(1)–C(1)–O(1) ip bend		241 m	242 s	CCl3 asym bend
	249 m	246 s	CCl3 asym bend		256 sh	255 s	CCl3 asym bend
		254 s	CCl3 asym bend			268 m	CCl3 asym bend
					326 w	326 m	C(1)–O(1)–C(2) + C(2)–O(2)–C(3) oop bend
	337 m	338 s	C(1)–O(1)–C(2) ip bend		355 w	357 m	C(1)–O(1)–C(2) + C(2)–O(2)–C(3) ip bend
					363 w	361 m	CCl3 oop sym bend
		390 s	O(1) oop bend		384 m	383 m	Skeletal deformation
					402 sh	402 m	Skeletal deformation
	401 vs	401 s	CCl3 sym bend		415 vs	413 s	CCl3 ip sym bend
495 s	498 vs	494 s	Cl(1)–C(1) = O(2) bend				
584 s	589 w	589 vs	CCl3 sym stretch			530 m	CCl3 oop sym stretch
				676 s	678 s	684 m	CCl3 ip sym stretch
665 m		671 s	C(1) oop bend				
759 s	764 w	770 w	CCl3 asym stretch			753 m	CCl3 asym stretch
807 vs	817 w	821 m	CCl3 asym stretch	806 vs,br			CCl3 asym stretch
					823 w	827 m	CCl3 asym stretch
					891 vw	891 w	CCl3 asym stretch
908 s/925 sh	915 w	917 w,br	O(1)–C(1) stretch	914 vs			C(1)–O(1) + O(3)–C(3) out-of-phase stretch
					957 w/972 w	963 w	C(1)–O(1) + O(3)–C(3) ip stretch
965 vs		965 w	Cl(1)–C(1) stretch				
1042 vs,br		1069 w	C(1)–O(1) stretch	1109 w	1103 vw		O(1)–C(2) + C(2)–O(3) ip stretch
				1171 vs,br			O(1)–C(2) + C(2)–O(3) oop stretch
1800 vs	1803 w		C(1) = O(2) stretch	1818 s,br	1819 w		C(2)= O(2) stretch

a w = weak, m = medium, s = strong, v = very, br = broad, sh = shoulder atom numbering: diphosgene Cl(1)–C(1)­(=O(2))–O(1)–C(2)­Cl3 and triphosgene Cl3C(1)–O(1)–C(2)­(=O(2))–O(3)–CCl3.

The literature on the vibrations of the trichloromethyl group is very sparse. The standard texts^49–51^ on the assignment of organic molecules generally only consider monohaloalkanes and the conformational isomerism that can result. They either only mention the C–Cl stretching modes of the–CCl_3_ group^50,51^ or do not discuss them at all.^49^ Table 5 shows a compilation of the available data. Even with this limited selection, some conclusions are possible. While each of the modes occurs in a different region, the large standard deviations for all of the modes, except for the asymmetric bend, show that these vibrations are not good group frequency modes. The intensities largely follow expected patterns: the symmetric modes are strong in the Raman and weak in the infrared and vice versa for the asymmetric modes, although there are exceptions, even in this small data set.

**Table 5:** Observed Infrared, Raman and INS Modes [cm^–1^] of the Trichloromethyl, CCl_3_, Group

	sym^a^ stretch				asym stretch				sym bend				asym bend				rock			
	cm^–1^	IR	R	INS	cm^–1^	IR	R	INS	cm^–1^	IR	R	INS	cm^–1^	IR	R	INS	cm^–1^	IR	R	INS
HCCl_3_ ^52–54^	670	s	s	w	756	vs	w	w	368	vw	s	w	258/268	w	s	w				
hexachloroethane^55^	432	no	vs	n/a	780	vs	no	n/a	288	no	vs	n/a	276	w	no	n/a	167	m	no	n/a
trichloroacetic acid^56–59^	459	m	vs	n/a	830/704	vs	m	n/a	283	n/a	s	n/a	280	n/a	m	n/a	209/218	n/a	s	n/a
diphosgene	589	s	w	vs	770/821	s	w	m	401	n/a	vs	s	246/254	n/a	m	s	117–144	n/a	m	s
triphosgene	530/684	s	s	m	753–891	s	w	m	415	n/a	vs	s	242–268	n/a	m	s	74–62	n/a	m	s
mean	561				788				351				262				156			
standard deviation	106				58				56				14				50			

a sym = symmetric; asym = asymmetric; IR, R and INS are the infrared, Raman and INS intensities; no. = not observed; n/a = not available (outside of spectral range of the instrument or not measured).

## Conclusion

In this work we present a comprehensive structural, spectroscopic, and thermodynamic study of diphosgene and triphosgene. A new polymorph of diphosgene (β-diphosgene) was discovered and structurally characterized, complementing the previously known α-form. Differential scanning calorimetry revealed unusual cold crystallization behavior, a phenomenon rarely observed in small organic molecules. Solid-state quantum chemical calculations reproduced the experimental lattice parameters with high accuracy and provided insight into the relative stability of the two polymorphs. The calculations rationalize the observed phase behavior, showing that β-diphosgene is favored at temperatures between 200 and 50 K. Infrared, Raman, and inelastic neutron scattering spectra of both diphosgene and triphosgene were measured and fully assigned with the aid of periodic DFT calculations. These data provide the first comprehensive solid-state vibrational characterization of these compounds.

## Supplementary Material



## Notes

Supporting Information crystallographic data, additional crystal structure pictures, Hirshfeld surface analysis, details on quantum chemical calculations and vibrational spectroscopy (PDF) cif file of β-diphosgene at 200 K (CIF) cif file of β-diphosgene at 100 K (CIF) cif file of triphosgene at 100 K (CIF) (PDF)
