# Supplementary material for: Structural, Thermodynamic, and Spectroscopic Characterization of Diphosgene and Triphosgene
Source: Inorg Chem. 2026 Mar 20;65(13):7071–81. doi: 10.1021/acs.inorgchem.5c05882 (PMC13058871; doi:10.1021/acs.inorgchem.5c05882)
Supplement: Supplementary file 1 [file ic5c05882_si_001.pdf]

Supporting Information

Structural, Thermodynamic, and Spectroscopic Characterization of Diphosgene and Triphosgene

Sven Ringelband[1], Stewart F. Parker[2] and Frank Tambornino*[1]

[1]*Department of Chemistry, Philipps-University Marburg, Marburg, Germany*

[2]ISIS Neutron and Muon Source, STFC Rutherford Appleton Laboratory, Chilton OX11 0QX, United Kingdom

(E-mail: frank.tambornino@chemie.uni-marburg.de)

Contents

1. Crystallographic data ... 2
   1.1. Additional pictures for triphosgene ... 3
   1.2. Additional pictures for β-diphosgene ... 4
2. Additional details on Hirshfeld surface analysis ... 6
   2.1. Triphosgene at 100 K ... 6
   2.2. β-Diphosgene at 200 K ... 7
   2.3. β-Diphosgene at 100 K ... 9
   2.4. α-Diphosgene literature ... 11
3. Differential scanning calorimetry of diphosgene ... 12
4. Details on quantum chemical calculations ... 14
5. Details on inelastic neutron scattering ... 15
   5.1. Vibrational analysis ... 15
6. References ... 27

1. Crystallographic data

Table S1: Selected single crystal X-ray data collection and refinement parameters for triphosgene at 100 K and β-diphosgene at 100 K and 200 K.

	Triphosgene	β-Diphosgene	β-Diphosgene
Formula	C3O3Cl6	$C_2O_2Cl_4$	$C_2O_2Cl_4$
CCDC	2516310	2516311	2516312
F. w. / g mol^{-1}	296.73	197.82	197.82
Crystal system	monoclinic	monoclinic	monoclinic
Space group	$P2_1/c$	$P2_1/n$	$P2_1/n$
a / Å	9.7241(7)	11.6703(3)	11.7485(2)
b / Å	8.7991(7)	7.3870(2)	7.4109(2)
c / Å	11.1583(9)	15.3222(4)	15.4942(6)
α / °	90	90	90
β / °	91.330(6)	94.574(2)	94.581(3)
γ / °	90	90	90
V / Å^3	954.48(13)	1316.70(6)	1344.72(9)
Z	4	8	8
Radiation, λ / Å	1.54186	1.54186	1.54186
Temp / K	100	100	200
ρ_{calc} / g cm^{-3}	2.065	1.996	1.954
μ / mm^{-1}	15.32	15.64	15.64
Reflections collected	7356	19134	21905
Ind. Reflns. / Ind. Reflns gt	1763 / 1512	2436 / 2287	2486 / 2066
Parameters	109	145	145
R_{int} / $R_{(\sigma)}$ / %	4.43 / 2.86	8.39 / 3.59	8.80 / 4.13
$R1/wR2$,[a] $I \geq 2\sigma I$ / %	4.78 / 12.78	6.35 / 18.02	6.07 / 17.47
$R1/wR2$,[a] all data / %	5.65 / 13.49	6.56 / 18.34	6.83 / 17.90
GOF	1.066	1.149	1.074

[a] $R1 = [\Sigma||F_o| - |F_c||]/\Sigma|F_o|$; $wR2 = \{[\Sigma w[(F_o)^2 - (F_c)^2]^2]/[\Sigma w(F_o^2)^2]\}^{1/2}$; $w = [\sigma^2(F_o)^2 + (AP)^2 + BP]^{-1}$, where $P = [(F_o)^2 + 2(F_c)^2]/3$ and the A and B values are 0.077100 and 1.893900 for triphosgene, 0.144100 and 1.046500 for β-diphosgene at 100 K, 0.123800 and 0.00 for β-diphosgene at 200 K.

1.1. Additional pictures for triphosgene

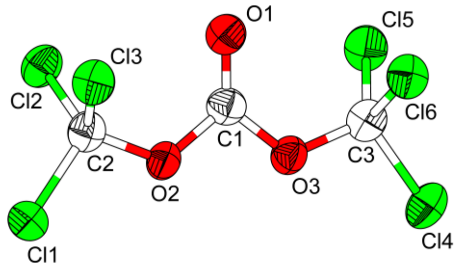

Figure S1: Molecular structure of triphosgene in the single crystal. Ellipsoids are drawn with 60% displacement at 100 K. Colour code: O red, C white, Cl green.

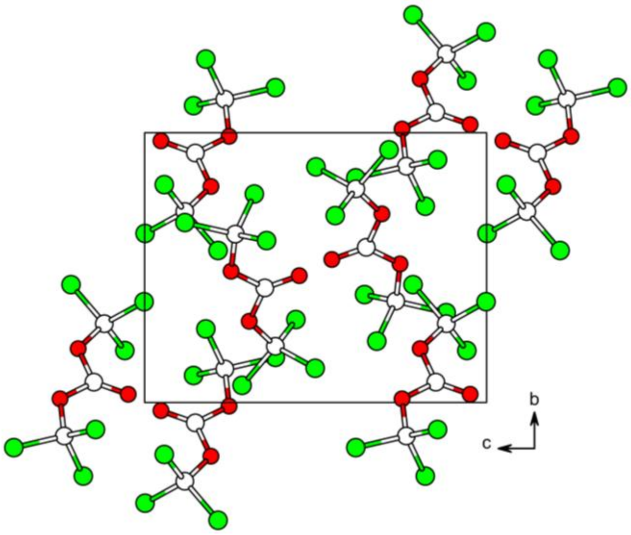

Figure S2: Crystal structure of triphosgene at 100 K viewed along [100]. Atoms are depicted with arbitrary radii. Colour code: O red, C white, Cl green.

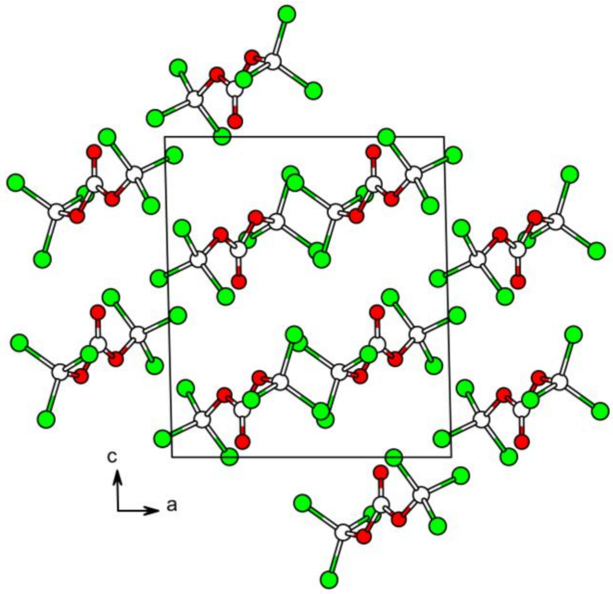

Figure S3: Crystal structure of triphosgene at 100 K viewed along [010]. Atoms are depicted with arbitrary radii. Colour code: O red, C white, Cl green.

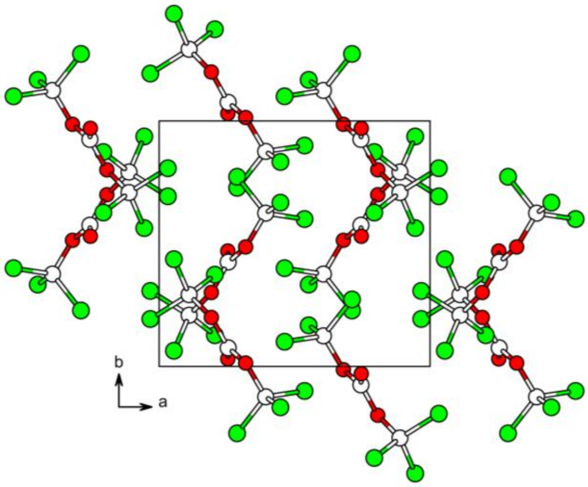

Figure S4: Crystal structure of triphosgene at 100 K viewed along [001]. Atoms are depicted with arbitrary radii. Colour code: O red, C white, Cl green.

1.2. Additional pictures for β-diphosgene

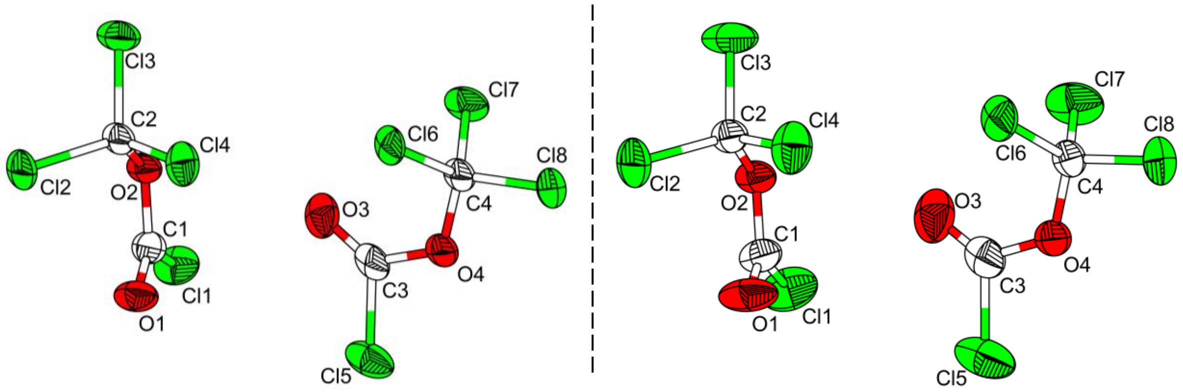

Figure S5: Molecular structure of β-diphosgene in the single crystal. Ellipsoids are drawn with 60% displacement at 100 K (left) and 200 K (right). Colour code: O red, C white, Cl green.

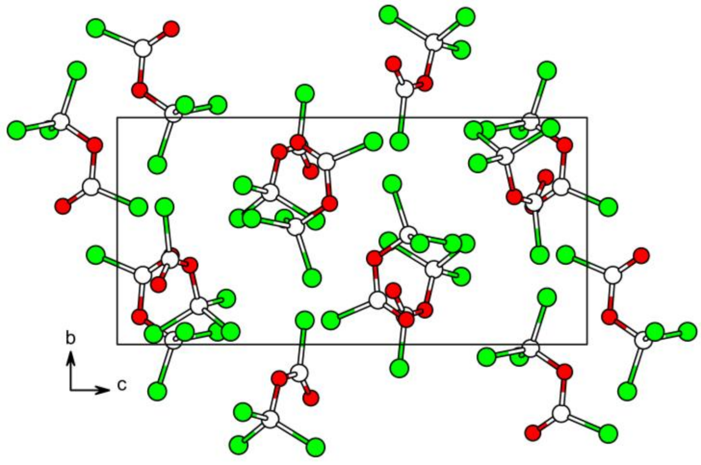

Figure S6: Crystal structure of β-diphosgene at 100 K viewed along [100]. Atoms are depicted with arbitrary radii. Colour code: O red, C white, Cl green.

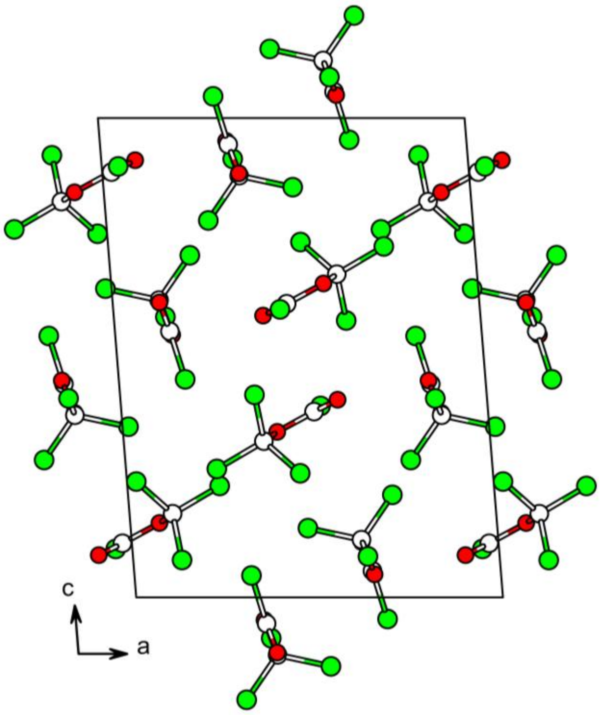

Figure S7: Crystal structure of β-diphosgene at 100 K viewed along [010]. Atoms are depicted with arbitrary radii. Colour code: O red, C white, Cl green.

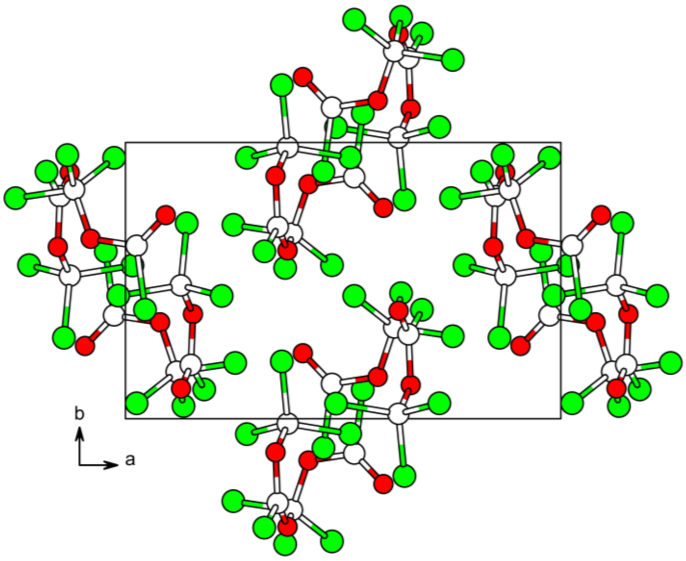

Figure S8: Crystal structure of β-diphosgene at 100 K viewed along [001]. Atoms are depicted with arbitrary radii. Colour code: O red, C white, Cl green.

2. Additional details on Hirshfeld surface analysis

2.1. Triphosgene at 100 K

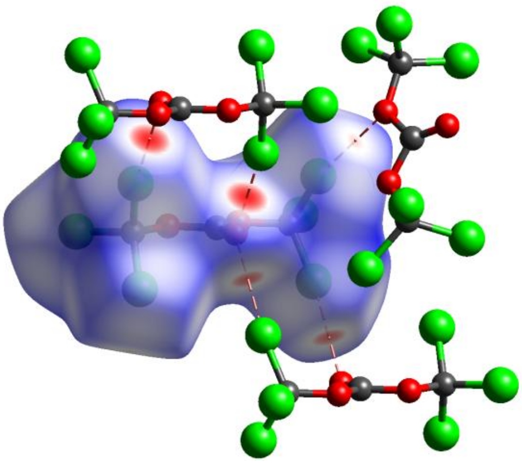

Figure S9: Hirshfeld surface of triphosgene shown with neighbouring molecules. Red areas indicate short contacts. Red dashed lines highlight O···Cl short contacts. Colour code: O red, C grey, Cl green.

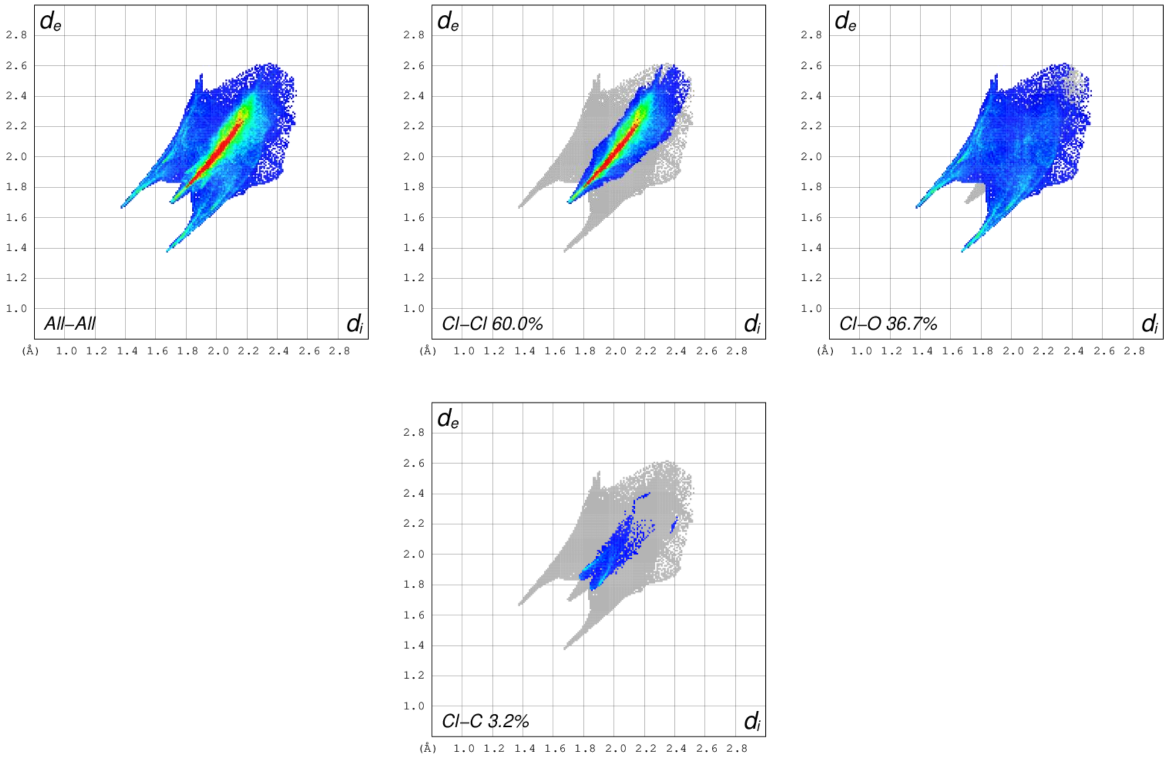

Figure S10: Fingerprint plots for triphosgene mapped from the Hirshfeld surface. Surface contacts and overall quantity are provided in each graph.

2.2. β-Diphosgene at 200 K

Molecule 1

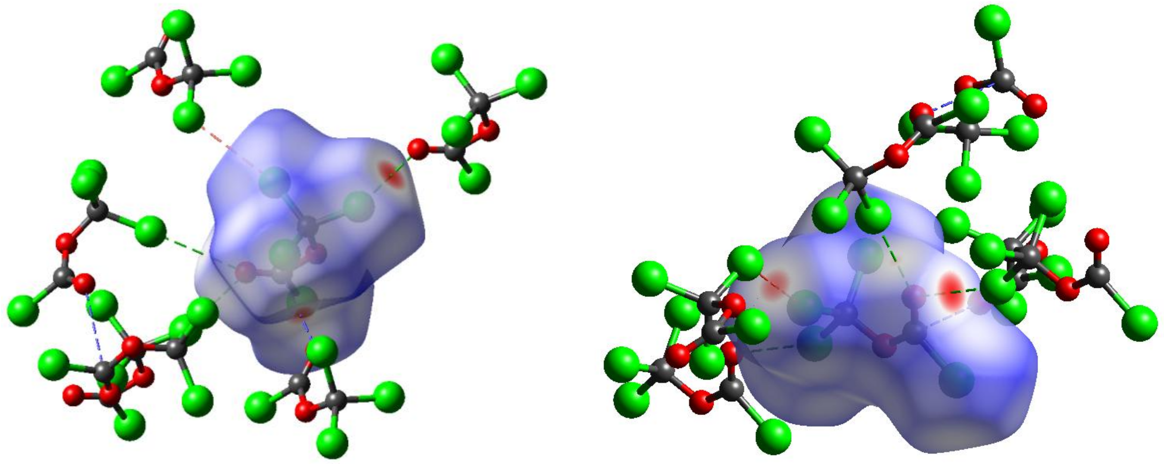

Figure S11: Hirshfeld surface of β-diphosgene at 200 K shown with neighbouring molecules. Two different viewing directions. Red areas indicate short contacts. Red dashed lines highlight Cl···Cl and green dashed lines the Cl···O short contacts and blue C···O short contacts. Colour code: O red, C grey, Cl green.

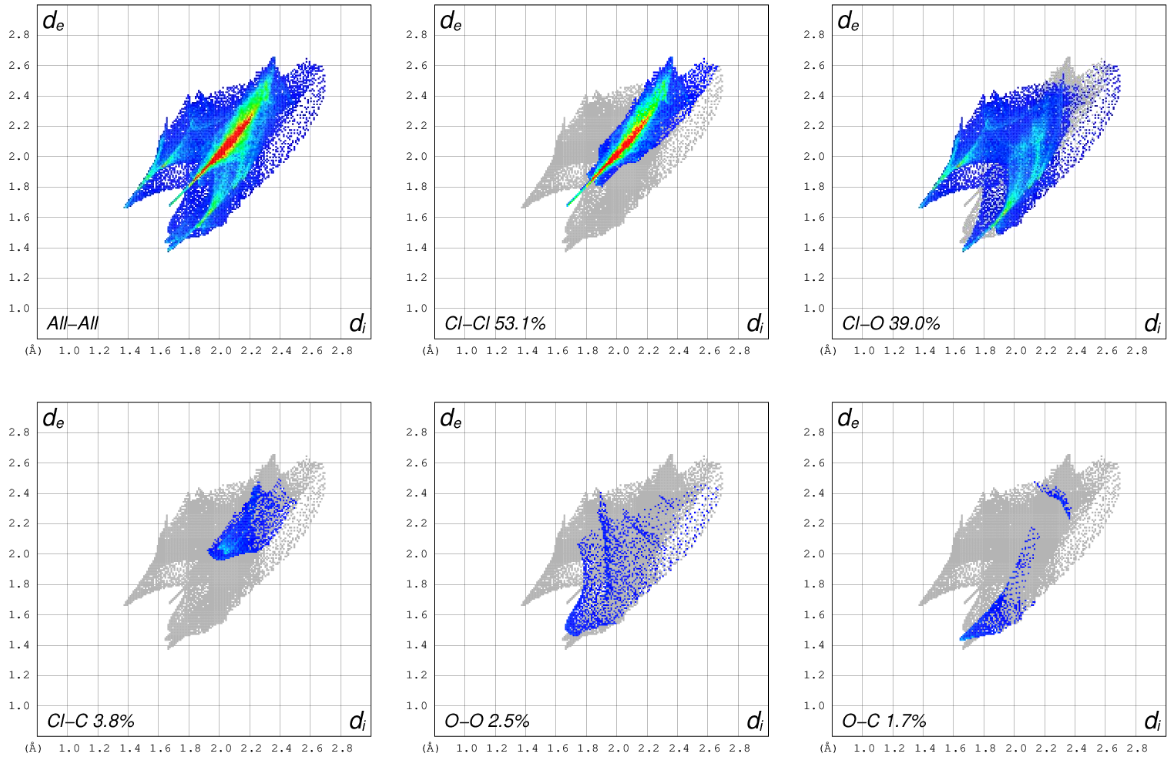

Figure S12: Fingerprint plots for β-diphosgene at 200 K mapped from the Hirshfeld surface. Surface contacts and overall quantity are provided in each graph.

Molecule 2

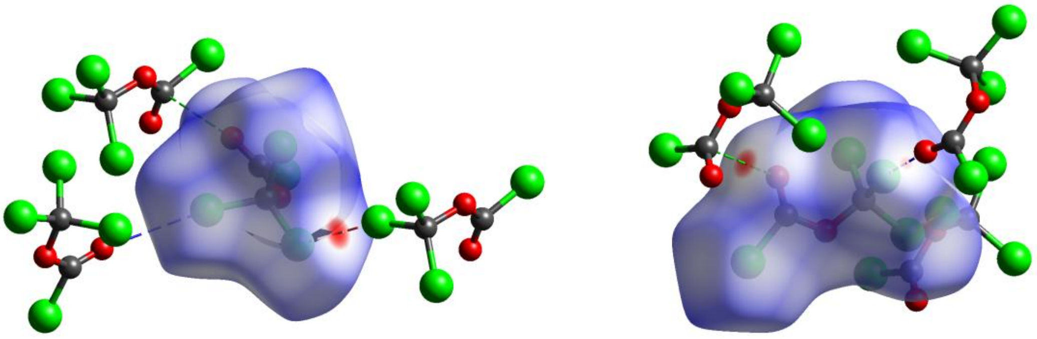

Figure S13: Hirshfeld surface of β-diphosgene at 200 K shown with neighbouring molecules. Two different viewing directions. Red areas indicate short contacts. Red dashed lines highlight Cl···Cl, green the O···C and blue the O···Cl short contacts. Colour code: O red, C grey, Cl green.

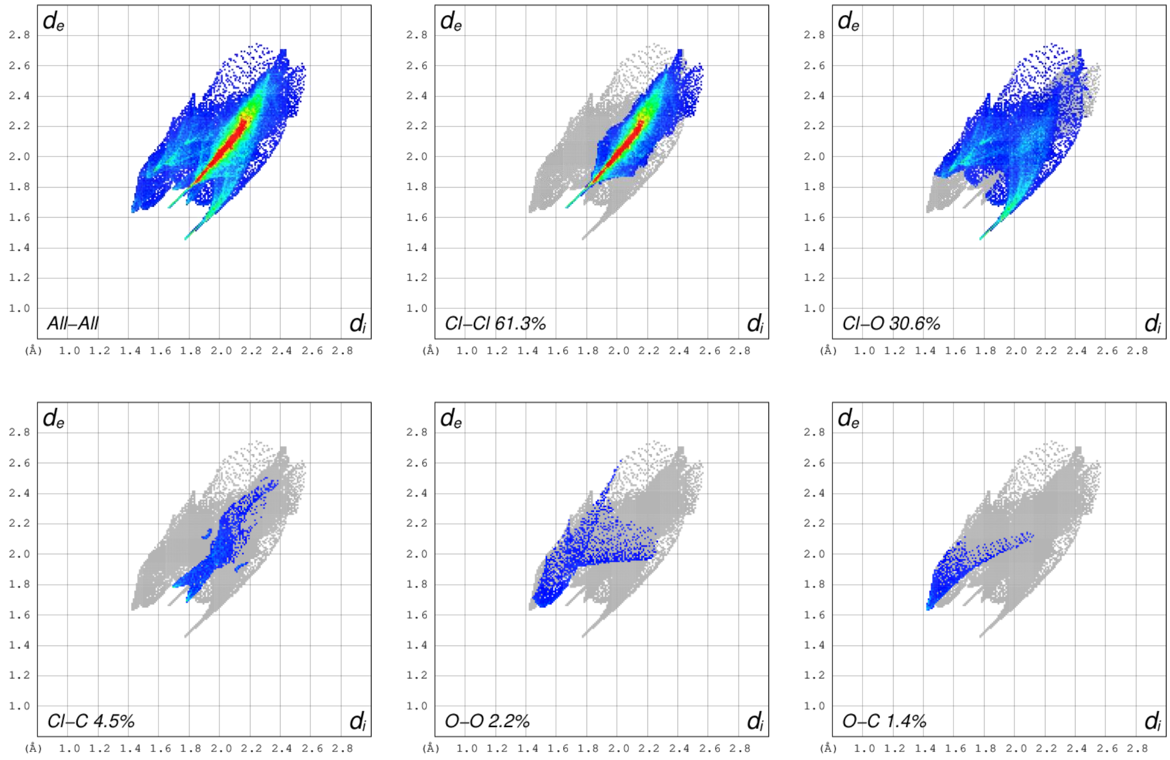

Figure S14: Fingerprint plots for β-diphosgene at 200 K mapped from the Hirshfeld surface. Surface contacts and overall quantity are provided in each graph.

2.3. β-Diphosgene at 100 K

Molecule 1

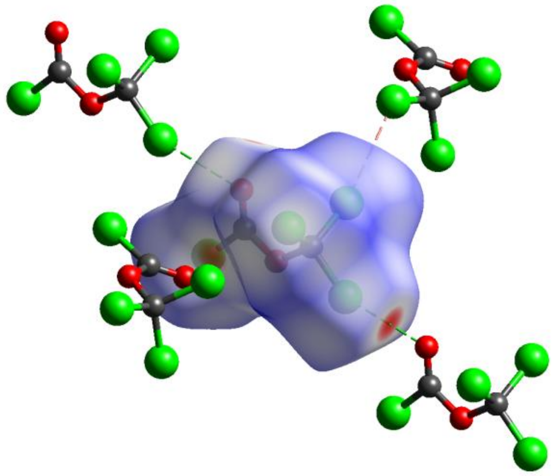

Figure S15: Hirshfeld surface of β-diphosgene at 100 K shown with neighbouring molecules. Red areas indicate short contacts. Red dashed lines highlight Cl···Cl and green dashed lines highlight O···Cl short contacts. Colour code: O red, C grey, Cl green.

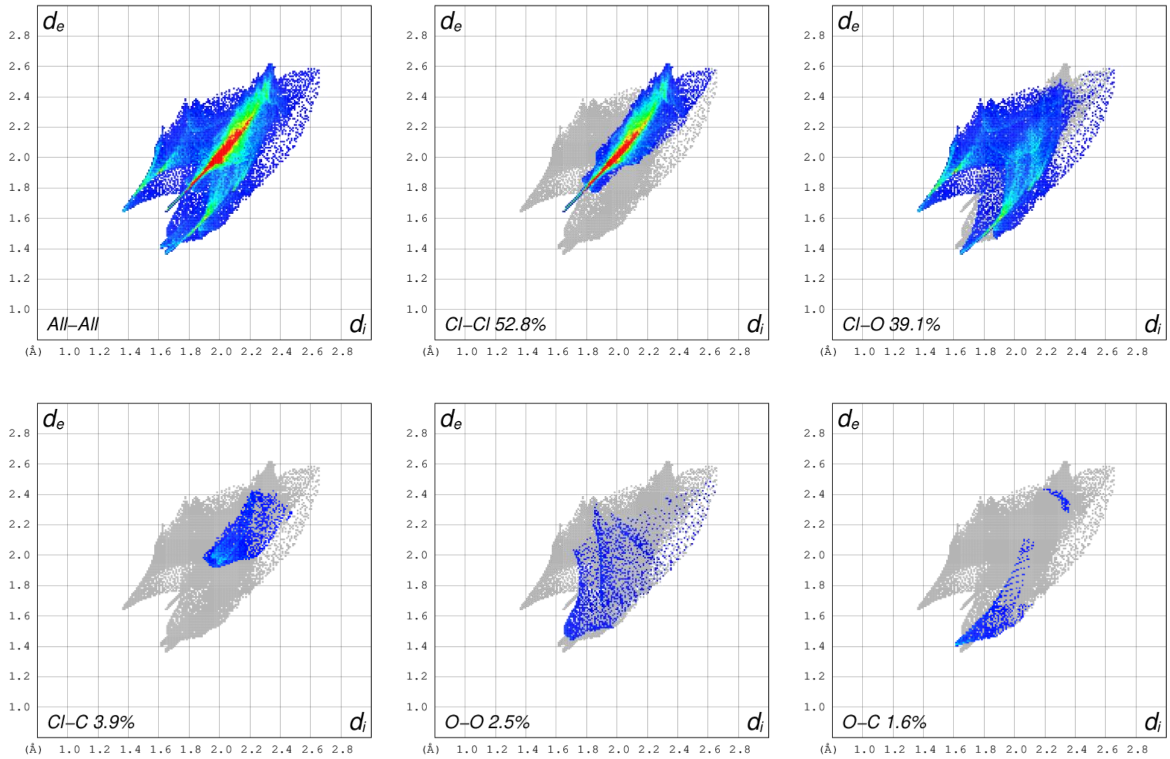

Figure S16: Fingerprint plots for β-diphosgene at 100 K mapped from the Hirshfeld surface. Surface contacts and overall quantity are provided in each graph.

Molecule 2

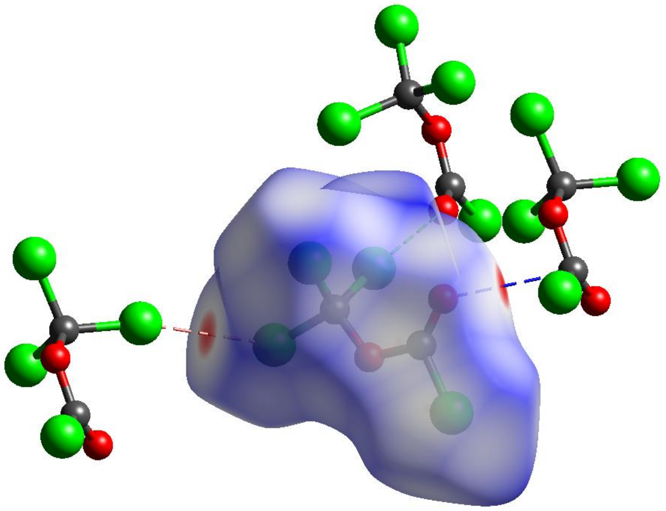

Figure S17: Hirshfeld surface of β-diphosgene at 100 K shown with neighbouring molecules. Red areas indicate short contacts. Red dashed lines highlight Cl···Cl, blue the O···C, and green the O···Cl short contact. Colour code: O red, C grey, Cl green.

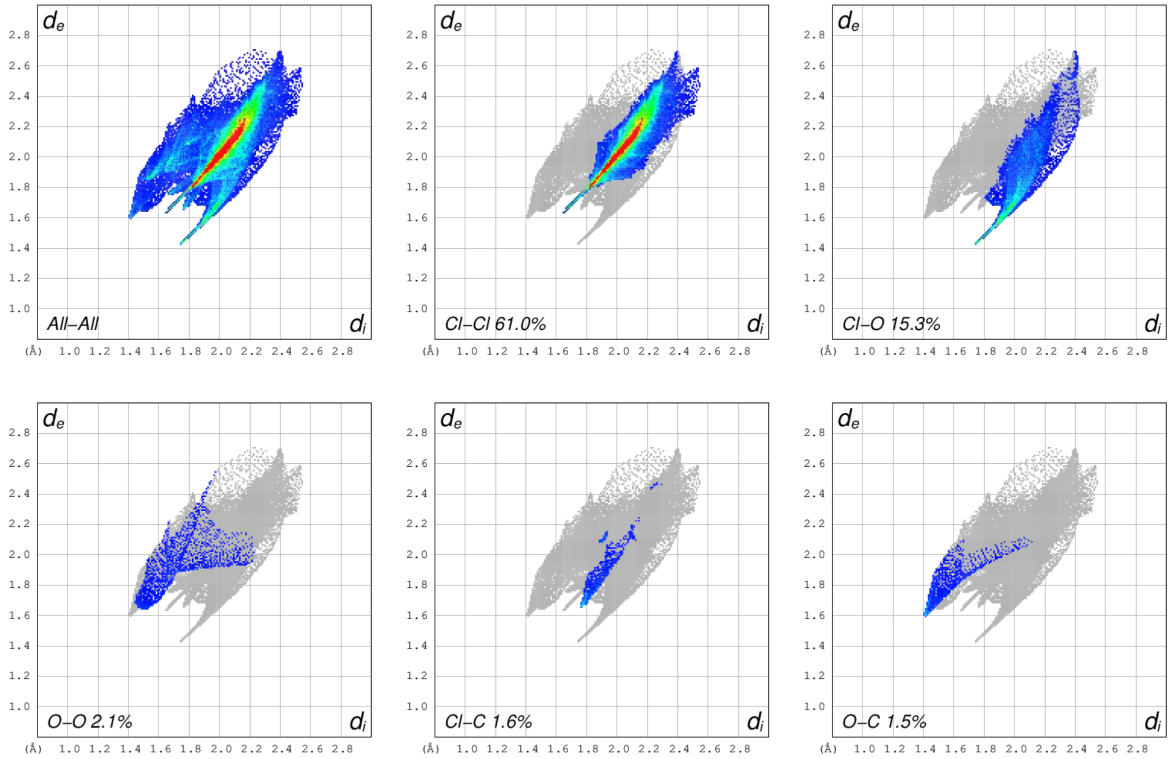

Figure S18: Fingerprint plots for β-diphosgene at 100 K mapped from the Hirshfeld surface. Surface contacts and overall quantity are provided in each graph.

2.4. α-Diphosgene literature

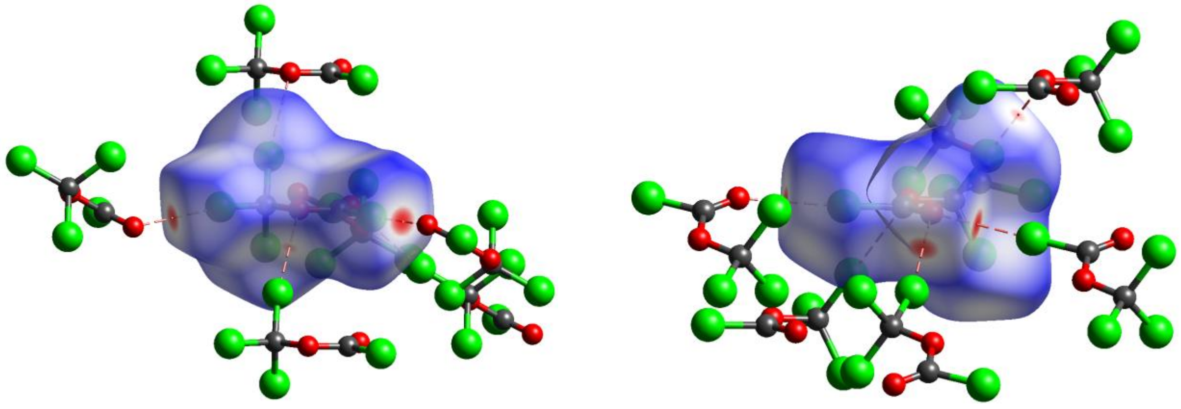

Figure S19: Hirshfeld surface of α-diphosgene shown with neighbouring molecules.[1] Two different viewing directions. Red areas indicate short contacts. Red dashed lines highlight O···Cl short contacts. Colour code: O red, C grey, Cl green.

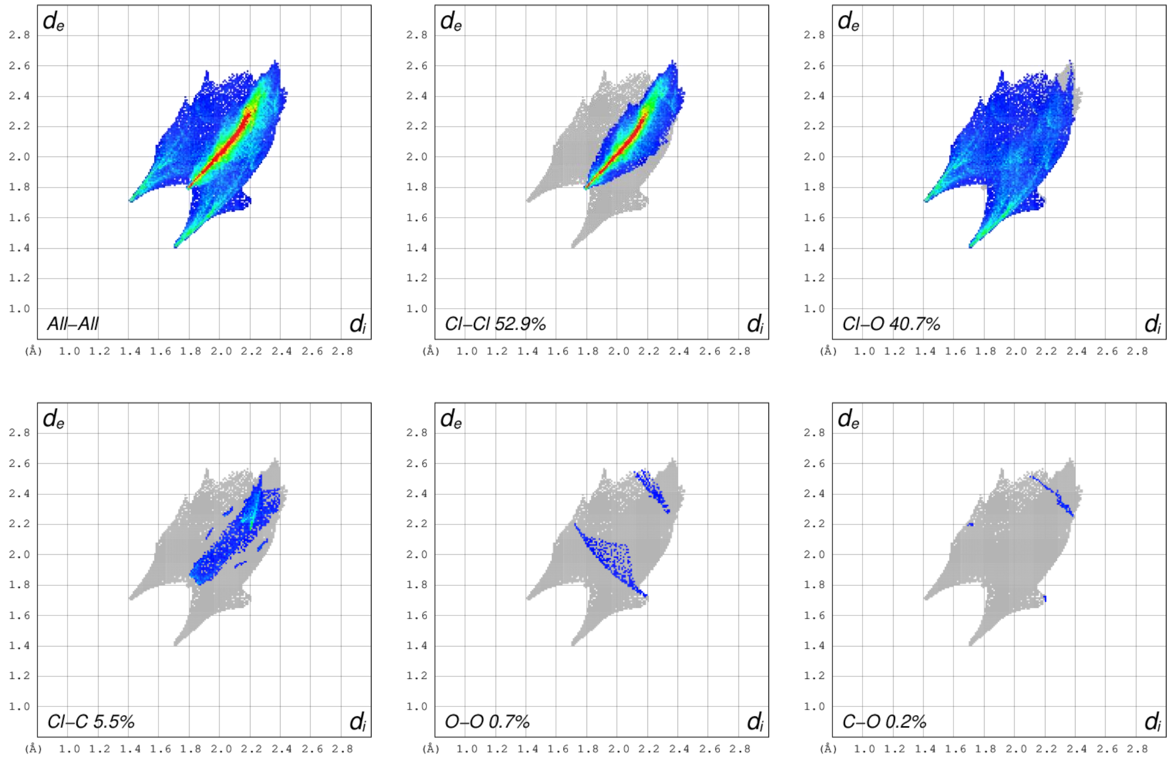

Figure S20: Fingerprint plots for α-diphosgene[1] mapped from the Hirshfeld surface. Surface contacts and overall quantity are provided in each graph.

3. Differential scanning calorimetry of diphosgene

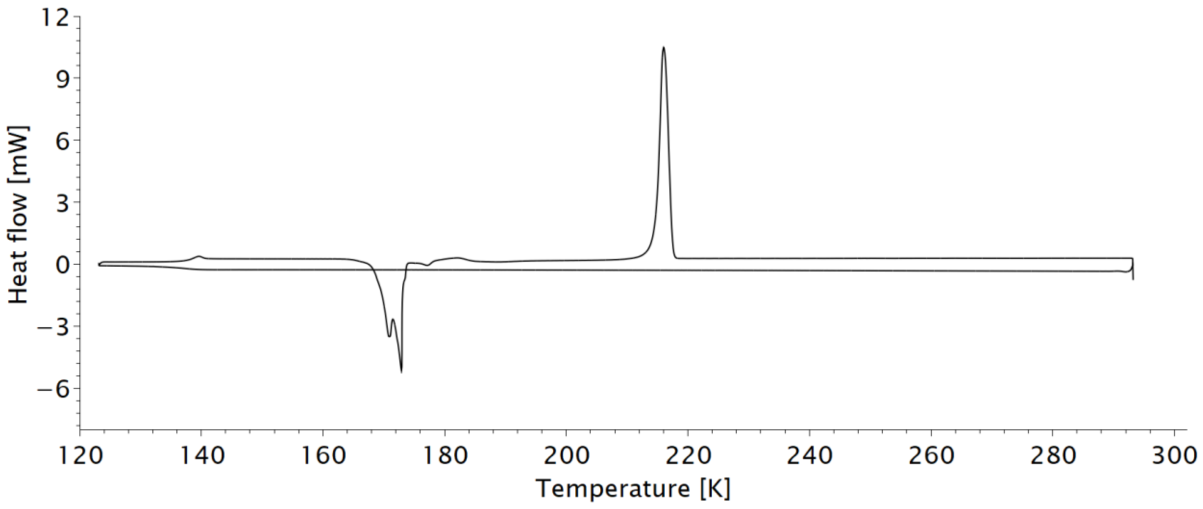

Figure S21: DSC curve of diphosgene at a cooling/heating rate of 10 K min^{-1}.

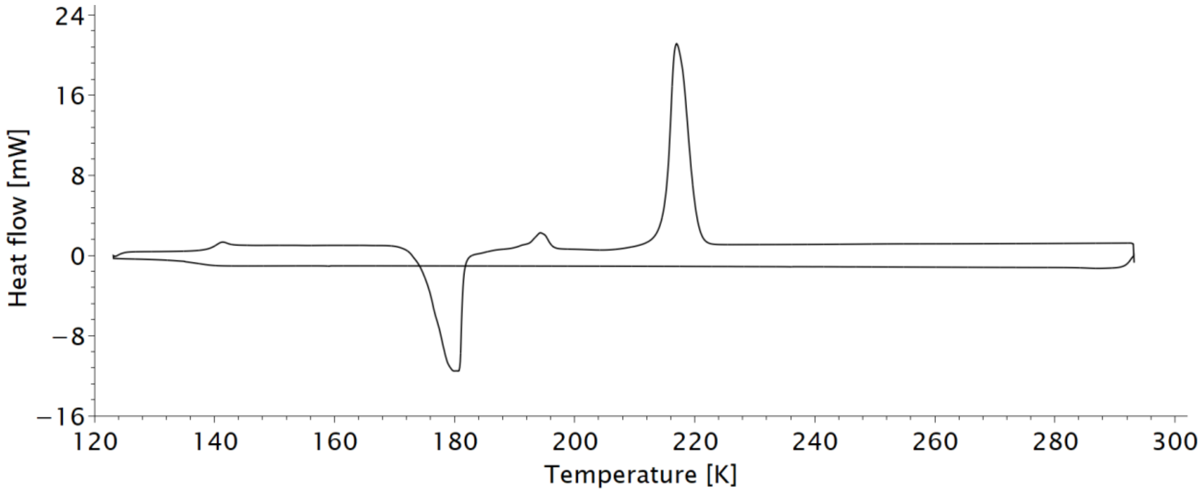

Figure S22: DSC curve of diphosgene at a cooling/heating rate of 4 K min^{-1}.

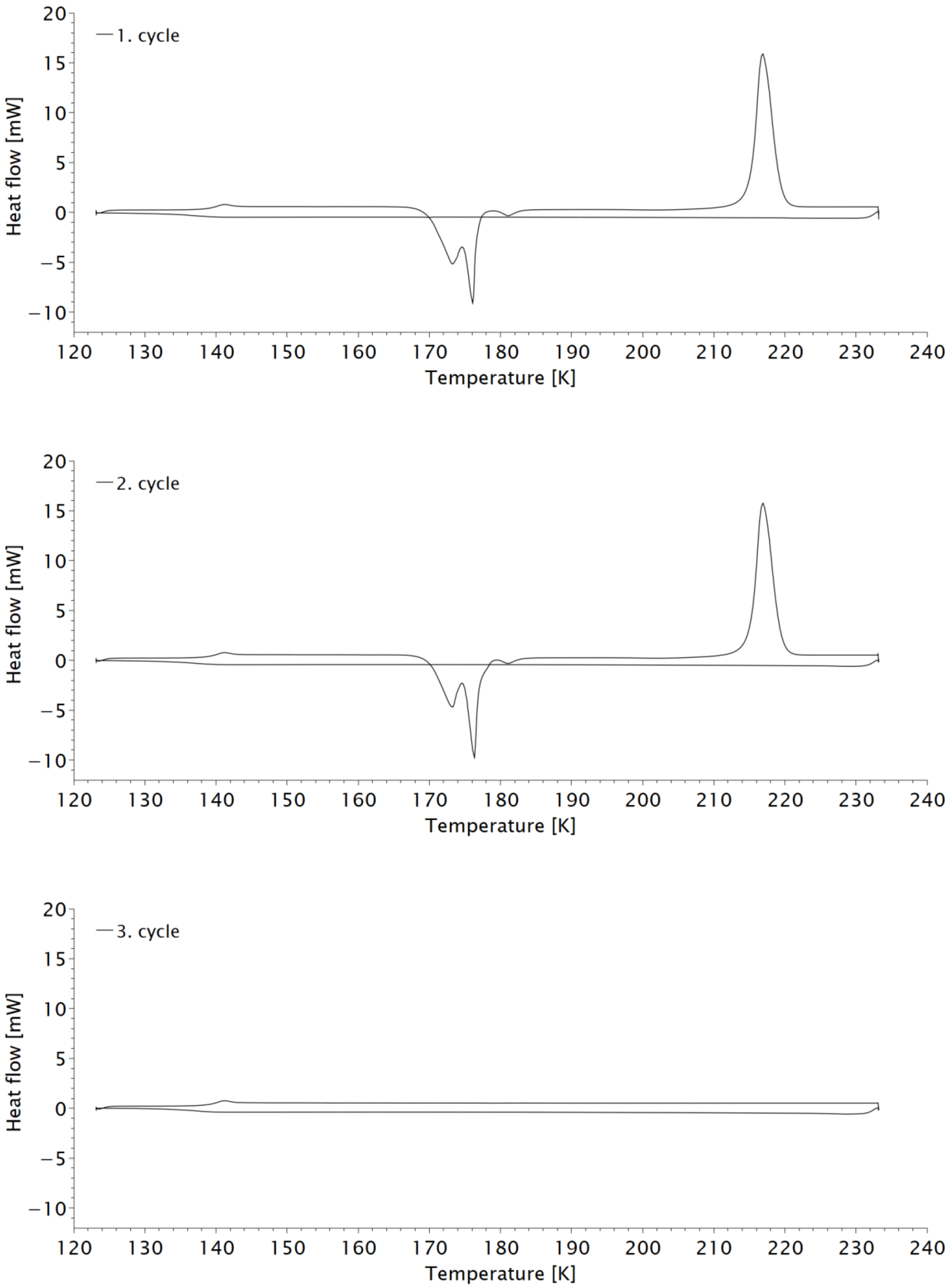

Figure S23: DSC hysteresis of three subsequent measurements of diphosgene at a cooling/heating rate of 4 K min^{-1}.

4. Details on quantum chemical calculations

Table S2: Lattice parameters and atomic coordinates of the optimized solid-state structure of triphosgene in space group $P2_1/c$. Level of theory: DFT-PBE0-D3(BJ-ABC)/def2-TZVP.

Lattice parameter	*a* [Å]	*b* [Å]	*c* [Å]	*β* [°]
	9.49256028	8.72860377	11.07634618	91.981841

Atom	*x*	*y*	*z*
Cl1	0.45424	0.40312	0.35594
Cl2	0.04170	–0.30639	0.44059
Cl3	–0.29099	0.22614	0.32197
Cl4	–0.19968	–0.12265	–0.49870
Cl5	–0.05396	–0.06093	0.28125
Cl6	–0.45350	0.33695	0.11472
O7	–0.19279	–0.29916	0.30923
O8	–0.32189	–0.48671	0.25614
O9	–0.25298	–0.46859	0.45453
C10	–0.25575	–0.42404	0.35374
C11	–0.39790	0.37786	0.26542
C12	–0.10719	–0.20570	0.38169

Table S3: Lattice parameters and atomic coordinates of the optimized solid-state structure of β-diphosgene in space group $P2_1/n$. Level of theory: DFT-PBE0-D3(BJ-ABC)/def2-TZVP.

Lattice parameter	*a* [Å]	*b* [Å]	*c* [Å]	*β* [°]
	11.49919100	7.35195443	14.96028412	92.970999

Atom	*x*	*y*	*z*
Cl1	–0.47469	0.43127	–0.25131
Cl2	–0.01672	0.46843	–0.35398
Cl3	0.22602	0.49815	–0.29154
Cl4	–0.36503	0.44952	–0.42155
Cl5	0.13409	–0.26831	–0.43282
Cl6	–0.24492	0.27808	–0.26850
Cl7	0.18907	0.11448	0.45701
Cl8	0.44901	–0.11304	–0.39805
O9	–0.42262	0.14347	–0.34550
O10	0.15794	0.39631	–0.45418
O11	0.13427	0.13572	–0.37692
O12	0.40341	0.23302	–0.40823
C13	0.15618	0.21540	–0.44220
C14	0.47088	0.11972	–0.38531
C15	0.12714	–0.48725	–0.38563
C16	–0.38219	0.31799	–0.32396

Table S4: Lattice parameters and atomic coordinates of the optimized solid-state structure of literature α-diphosgene in space group $P2_1/n$. Level of theory: DFT-PBE0-D3(BJ-ABC)/def2-TZVP.

Lattice parameter	*a* [Å]	*b* [Å]	*c* [Å]	*β* [°]
	5.45364496	14.09160927	8.46228490	103.715801

Atom	*x*	*y*	*z*
Cl1	0.30291	–0.21994	0.45160
Cl2	0.21289	0.07218	–0.25226
Cl3	–0.22799	–0.04885	–0.29921
Cl4	0.23199	–0.09609	–0.05622
C5	0.15626	–0.18533	–0.39773
C6	0.10576	–0.04595	–0.24989
O7	0.03924	–0.23611	–0.33233
O8	0.20078	–0.09204	–0.36930

5. Details on inelastic neutron scattering

5.1. Vibrational analysis

The correlation tables[2] for α- and β-diphosgene and that for triphosgene are given in Tables S5-S7. In the crystal, α-, β-diphosgene and triphosgene occupy C_1 sites, with the factor group being C_{2h}. The C_1 site removes all symmetry, so the only representation is *A*, which under C_{2h} symmetry, correlates to $A_g + A_u + B_g + B_u$.

Table S5: Correlation table for α-diphosgene, $C_2O_2Cl_4$, (space group $P2_1$/n, no. 14).

**Free molecule**		**Crystal**	
$\mathbf{C_s}$		**Site** [1] **(C_1)**	**Factor group (C_{2h}, Z = 4)**
**Rep.** [2]		**Rep.**	
External	Internal		
$2A' + A''$ (trans)		$3A$	$3A_g + 3A_u + 3B_g + 3B_u$
$A' + 2A''$ (lib)		$3A$	$3A_g + 3A_u + 3B_g + 3B_u$
	$12A'$	$12A$	$12A_g + 12A_u + 12B_g + 12B_u$
	$6A''$	$6A$	$6A_g + 6A_u + 6B_g + 6B_u$

[1] Symmetry of the site occupied by the ion in the crystal.

[2] Rep. = irreducible representation of the point group, trans. = translation, lib = libration.

Table S6: Correlation table for β-diphosgene, $C_2O_2Cl_4$, (space group $P2_1/n$, no. 14).

**Free molecule**		**Crystal**	
$\boldsymbol{C_s}$		**Site**[1] **($\boldsymbol{C_1}$)**	**Factor group ($\boldsymbol{C_{2h}}$, Z = 8)**
**Rep.**[2]		**Rep.**	
External	Internal		
$2A' + A''$ (trans)		$3A$	$6A_g + 6A_u + 6B_g + 6B_u$
$A' + 2A''$ (lib)		$3A$	$6A_g + 6A_u + 6B_g + 6B_u$
	$12A'$	$12A$	$24A_g + 24A_u + 24B_g + 24B_u$
	$6A''$	$6A$	$12A_g + 12A_u + 12B_g + 12B_u$

[1] Symmetry of the site occupied by the ion in the crystal.

[2] Rep. = irreducible representation of the point group, trans. = translation, lib = libration.

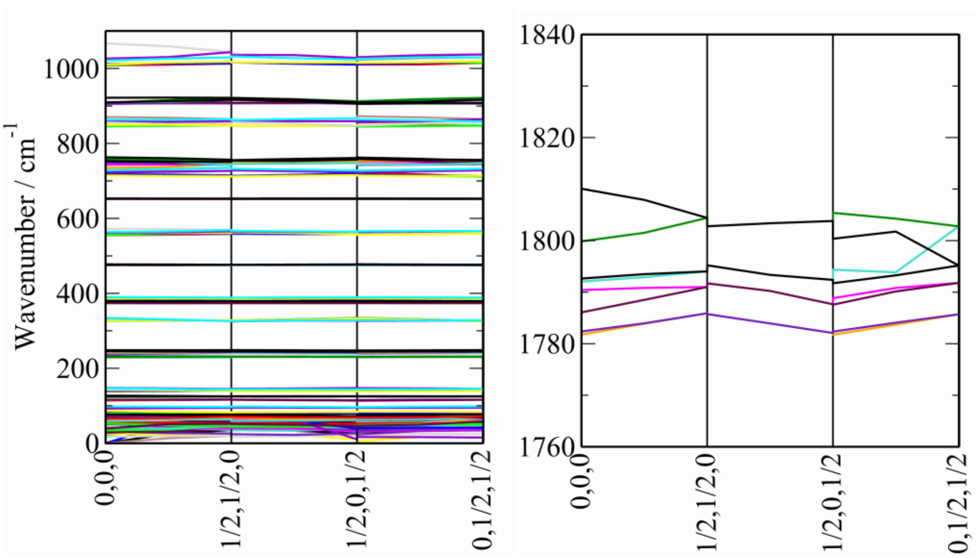

Figure S24: Dispersion curves of α-diphosgene.

Table S7: Calculated transition energies, infrared and Raman intensities at the Brillouin zone Γ-point for α-diphosgene.

Transition energy / cm^{-1}	Rep.[a]	Average[b] / cm^{-1}	Range[c] / cm^{-1}	Infrared intensity / km mol^{-1}	Raman intensity / $Å^4$ amu^{-1}	Assignment[d]
0	Bu	14	29	0.00	0.00	Acoustic & optic translation
0	Bu	25	47	0.00	0.00	Acoustic & optic translation
0	Au			0.00	0.00	
18	Au			0.04	0.00	
28	Bg			0.00	1.97	
29	Ag			0.00	2.81	
36	Ag			0.00	1.19	
41	Au	47	16	0.67	0.00	Optic translation
41	Ag			0.00	3.18	
47	Bg			0.00	1.19	
49	Bu			0.71	0.00	
52	Ag	56	9	0.00	2.31	Libration
54	Au			0.08	0.00	
55	Bu			1.10	0.00	
57	Au	63	11	0.00	0.00	Libration
57	Bg			0.00	0.38	
61	Bg			0.00	1.37	
63	Bg			0.00	4.13	
64	Ag			0.00	4.04	
68	Bu			0.23	0.00	
72	Bu	74	5	0.66	0.00	Libration
74	Au			1.37	0.00	
75	Ag			0.00	24.87	
77	Bg			0.00	3.98	
82	Bg	85	6	0.00	11.91	CCl3 torsion
84	Ag			0.00	19.79	
85	Bu			1.62	0.00	
88	Au			0.61	0.00	
112	Bg	116	10	0.00	26.32	CCl3 rock
115	Ag			0.00	5.60	
116	Au			0.89	0.00	
122	Bu			2.66	0.00	
134	Bu	136	4	3.58	0.00	CCl3 rock
136	Au			2.77	0.00	
137	Ag			0.00	42.08	
138	Bg			0.00	21.56	
231	Bu	233	4	3.12	0.00	Cl(1)-C(1)-O(1) ip bend

232	Ag			0.00	25.69	
233	Au			0.43	0.00	
235	Bg			0.00	26.92	
235	Ag	237	3	0.00	77.07	CCl3 asym bend
237	Bg			0.00	105.64	
237	Au			6.84	0.00	
238	Bu			2.31	0.00	
244	Ag	247	7	0.00	6.65	CCl3 asym bend
245	Bg			0.00	88.20	
250	Bu			0.01	0.00	
250	Au			1.10	0.00	
322	Bu	325	7	4.59	0.00	C(1)-O(2)-C(2) ip bend
324	Bg			0.00	70.40	
327	Ag			0.00	77.54	
328	Au			0.23	0.00	
382	Bu	383	2	4.91	0.00	O(2) oop bend
382	Au			0.83	0.00	
383	Ag			0.00	80.33	
384	Bg			0.00	20.00	
386	Bu	387	1	6.13	0.00	CCl3 sym bend
386	Bg			0.00	1.40	
387	Ag			0.00	252.38	
387	Au			0.07	0.00	
478	Au	480	5	10.27	0.00	Cl(1)-C(1)-O(1) bend
478	Bg			0.00	8.71	
482	Bu			10.08	0.00	
483	Ag			0.00	342.44	
563	Bu	567	8	596.34	0.00	CCl3 sym stretch
565	Ag			0.00	91.53	
569	Au			98.97	0.00	
571	Bg			0.00	24.81	
659	Ag	659	0	0.00	1.79	C(1) oop bend
659	Bg			0.00	1.77	
659	Bu			27.32	0.00	
659	Au			19.29	0.00	
711	Bg	721	24	0.00	10.39	CCl3 asym stretch
712	Ag			0.00	102.47	
727	Au			709.92	0.00	
735	Bu			570.82	0.00	

745	Ag	749	8	0.00	55.63	CCl3 asym stretch
747	Bg			0.00	6.47	
751	Au			185.69	0.00	
753	Bu			1128.26	0.00	
841	Bu	847	13	1971.38	0.00	Cl(1)-C(1) stretch
845	Ag			0.00	70.93	
850	Au			111.82	0.00	
854	Bg			0.00	21.19	
896	Ag	904	19	0.00	79.89	O(2)-C(2) stretch
900	Au			1178.25	0.00	
905	Bg			0.00	15.68	
915	Bu			54.41	0.00	
1017	Ag	1025	21	0.00	30.44	C(1)-O(2) stretch
1018	Au			1704.67	0.00	
1026	Bu			1807.06	0.00	
1038	Bg			0.00	3.90	
1767	Ag	1775	21	0.00	341.56	C(1)=O(1) stretch
1769	Bu			1500.39	0.00	
1776	Au			1249.54	0.00	
1787	Bg			0.00	109.38	

[a]Irreducible representation in C_{2h} symmetry.

[b]Average of the factor group components.

[c]Difference between the highest and lowest energy factor group components.

[d]Cl(1)-C(1)(=O(1))-O(2)-C(2)-Cl(2ip)-Cl(3oop)(Cl3'oop)

Table S8: Correlation table for triphosgene, $C_3O_3Cl_6$, (space group $P2_1/c$, no. 14).

**Free molecule**		**Crystal**	
$\boldsymbol{C_{2v}}$		**Site** [1] **($\boldsymbol{C_1}$)**	**Factor group ($\boldsymbol{C_{2h}}$, Z = 4)**
**Rep.** [2]		**Rep.**	
External	Internal		
$A_1 + 2B_1$ (trans)		$3A$	$3A_g + 3A_u + 3B_g + 3B_u$
$A_2 + 2B_2$ (lib)		$3A$	$3A_g + 3A_u + 3B_g + 3B_u$
	$10\ A_1$	$10A$	$10A_g + 10A_u + 10B_g + 10B_u$
	$5\ A_2$	$5A$	$5A_g + 5A_u + 5B_g + 5B_u$
	$6B_1$	$6A$	$6A_g + 6A_u + 6B_g + 6B_u$
	$9B_2$	$9A$	$9A_g + 9A_u + 9B_g + 9B_u$

[1] Symmetry of the site occupied by the ion in the crystal.

[2] Rep. = irreducible representation of the point group, trans = translation, lib = libration.

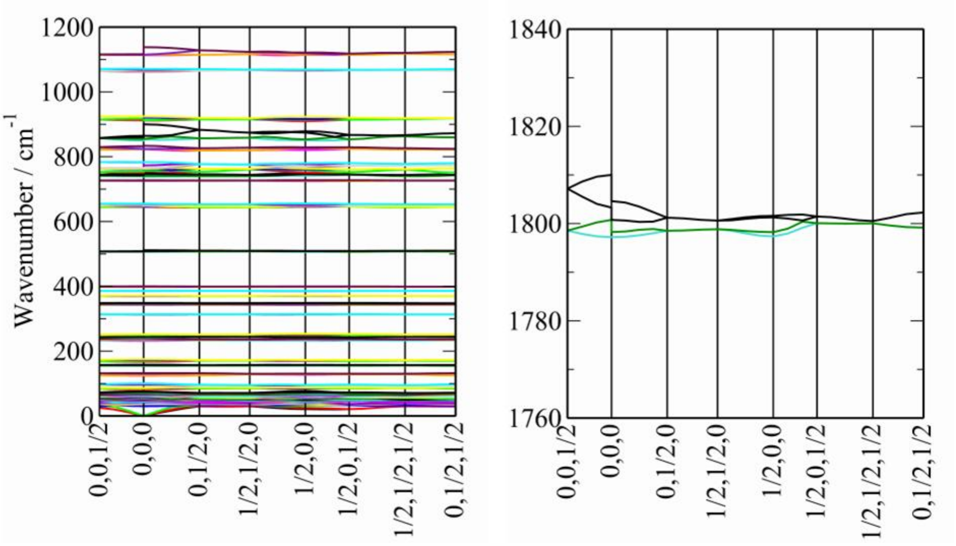

Figure S25: Dispersion curves of triphosgene. (The discontinuities at 0,0,0 are due to LOTO splitting).

Table S9: Calculated transition energies, infrared and Raman intensities at the Brillouin zone Γ-point for triphosgene.

Transition energy / cm^{-1}	Rep.[a]	Average[b] / cm^{-1}	Range[c] / cm^{-1}	Infrared intensity / km mol^{-1}	Raman intensity / $Å^4$ amu^{-1}	Assignment[d]
0	Au	18	36	0.00	0.00	Acoustic & optic translation
0	Bu			0.00	0.00	
0	Bu	28	43	0.00	0.00	Acoustic & optic translation
30	Au			0.54	0.00	
36	Ag			0.00	0.38	
36	Bg			0.00	0.29	
37	Bu	46	10	0.04	0.00	Optic translation
41	Bg			0.00	0.28	
43	Ag			0.00	0.39	
46	Ag			0.00	1.18	
47	Bu	52	8	0.26	0.00	Libration
47	Au			0.00	0.00	
51	Au			0.54	0.00	
53	Bg			0.00	2.32	
55	Bg			0.00	0.59	
55	Au	59	6	0.07	0.00	Libration
55	Ag			0.00	0.71	
57	Au	61	9	0.14	0.00	CCl3 antiphase torsion
58	Bu			1.10	0.00	
60	Bu			0.58	0.00	
60	Bg			0.00	0.42	
61	Bu	66	9	0.54	0.00	Libration
61	Ag			0.00	1.29	
63	Bg			0.00	0.22	
66	Ag			0.00	2.05	
66	Au			0.13	0.00	
66	Ag			0.00	0.47	
68	Bu	72	7	0.58	0.00	CCl3 rock
70	Ag			0.00	4.58	
70	Bg			0.00	1.68	
75	Au			0.20	0.00	
75	Bg			0.00	0.80	
82	Bg	85	4	0.00	2.32	CCl3 rock
85	Bu			0.41	0.00	
86	Ag			0.00	5.52	
86	Au			0.01	0.00	

96	Au	97	5	0.00	0.00	CCl3 rock
96	Bu			0.07	0.00	
97	Ag			0.00	23.86	
101	Bg			0.00	10.89	
124	Bg	128	7	0.00	1.66	O(2)C(2) + C(2)O(3) ip torsion
125	Ag			0.00	7.42	
131	Bu			9.38	0.00	
132	Au			0.61	0.00	
155	Ag	156	3	0.00	13.55	CCl3 rock
156	Bg			0.00	4.33	
156	Bu			1.77	0.00	
158	Au			1.96	0.00	
168	Au	171	7	0.58	0.00	O(1)C(2)O(3) bend
170	Ag			0.00	8.33	
173	Bu			0.53	0.00	
175	Bg			0.00	12.10	
231	Bg	233	2	0.00	23.31	CCl3 asym def
233	Au			0.02	0.00	
234	Bu			0.83	0.00	
234	Ag			0.00	32.25	
234	Au	237	6	0.30	0.00	CCl3 asym def
235	Ag			0.00	22.67	
237	Bu			0.61	0.00	
240	Bg			0.00	7.05	
241	Bu	243	3	0.51	0.00	CCl3 asym def
243	Bg			0.00	8.25	
244	Ag			0.00	15.67	
244	Au			0.02	0.00	
250	Au	251	2	6.34	0.00	CCl3 asym def
250	Bg			0.00	0.22	
252	Bu			2.69	0.00	
252	Ag			0.00	1.96	
313	Au	313	1	0.50	0.00	C(1)-O(1)-C(2) + C(2)-O(2)-C(3) out-of-phase bend
313	Ag			0.00	15.46	
313	Bg			0.00	23.42	
314	Bu			1.54	0.00	
343	Bg	344	1	0.00	41.13	C(1)-O(1)-C(2) + C(2)-

						O(2)-C(3) in-phase bend
344	Au			1.99	0.00	
344	Ag			0.00	25.82	
344	Bu			12.62	0.00	
347	Au	348	2	11.36	0.00	CCl3 oop sym bend
347	Bu			3.25	0.00	
348	Ag			0.00	23.96	
349	Bg			0.00	29.80	
370	Bg	370	2	0.00	26.40	Skeletal deformation
370	Au			1.77	0.00	
370	Bu			6.14	0.00	
371	Ag			0.00	84.24	
385	Bg	385	1	0.00	6.23	Skeletal deformation
385	Ag			0.00	17.51	
386	Bu			0.17	0.00	
386	Au			0.10	0.00	
398	Bu	399	2	1.66	0.00	CCl3 ip sym bend
399	Bg			0.00	17.61	
399	Au			0.51	0.00	
400	Ag			0.00	623.77	
507	Au	507	2	320.70	0.00	CCl3 out-of-phase sym stretch
507	Bu			189.75	0.00	
508	Bg			0.00	0.56	
508	Ag			0.00	2.55	
645	Bu	646	3	89.65	0.00	C=O ip bend
646	Bg			0.00	26.73	
646	Ag			0.00	10.00	
648	Au			181.18	0.00	
649	Ag	652	6	0.00	263.84	CCl3 in-phase sym stretch
649	Bu			418.69	0.00	
653	Au			69.12	0.00	
655	Bg			0.00	17.21	
726	Bg	726	1	0.00	1.06	C(2) out-of-plane deformation
726	Ag			0.00	1.87	
726	Bu			4.80	0.00	
727	Au			3.46	0.00	
739	Bu	742	9	2493.19	0.00	CCl3 asym stretch

741	Ag			0.00	24.23	
743	Au			1326.03	0.00	
747	Bg			0.00	10.03	
751	Au	758	12	187.88	0.00	CCl3 asym stretch
754	Ag			0.00	74.92	
762	Ag			0.00	30.12	
763	Bg			0.00	12.39	
764	Au	771	19	51.24	0.00	CCl3 asym stretch
765	Bu			141.98	0.00	
773	Bu			889.49	0.00	
783	Bg			0.00	22.67	
823	Bu	826	9	136.55	0.00	CCl3 asym stretch
824	Bg			0.00	45.42	
824	Ag			0.00	20.30	
832	Au			474.67	0.00	
850	Au	856	15	4046.27	0.00	C(1)-O(1) + O(3)-C(3) out-of-phase stretch
854	Bu			3014.60	0.00	
855	Bg			0.00	0.26	
864	Ag			0.00	7.86	
911	Bu	916	13	960.16	0.00	C(1)-O(1) + O(3)-C(3) in-phase stretch
912	Ag			0.00	77.01	
917	Au			79.43	0.00	
924	Bg			0.00	39.73	
1062	Ag	1067	9	0.00	111.73	O(1)-C(2) + C(2)-O(3) in-phase stretch
1068	Bg			0.00	2.48	
1068	Au			3.75	0.00	
1071	Bu			79.87	0.00	
1114	Bu	1114	1	1401.68	0.00	O(1)-C(2) + C(2)-O(3) out-of-phase stretch
1114	Au			2119.43	0.00	
1115	Ag			0.00	5.84	
1115	Bg			0.00	2.43	

1797	Ag	1800	6	0.00	199.08	C(2)=O(2) stretch
1798	Bu			2062.85	0.00	
1801	Bg			0.00	13.07	
1803	Au			222.42	0.00	

[a]Irreducible representation in C_{2h} symmetry.
[b]Average of the factor group components.
[c]Difference between the highest and lowest energy factor group components.
[d]Cl(2oop)(Cl2'oop)Cl(1ip)-C(1)-O(1)-C(2)(=O(2))-O(3)-C(3)-Cl(3ip)-Cl(4oop)(Cl4'oop)

Table S10: Calculated spectra of diphosgene with idealized C_s symmetry.

Transition energy / cm^{-1}	Rep.[1]	Infrared intensity / km mol^{-1}	Raman intensity / Å^4 amu^{-1}	Assignment[2]
72	A"	0.23	8.18	CCl3 rock
107	A"	0.12	1.95	CCl3 torsion
126	A'	0.67	1.68	CCl3 rock
225	A'	0.43	2.73	Cl(1)-C(1)-O(1) ip bend
230	A'	1.10	2.92	CCl3 asym bend
237	A"	0.12	1.39	CCl3 asym bend
322	A'	0.85	5.66	C(1)-O(2)-C(2) ip bend
374	A"	1.20	7.25	O(2) oop bend
381	A'	0.76	15.42	CCl3 sym bend
469	A'	8.55	17.58	Cl(1)-C(1)-O(1) bend
551	A'	167.66	15.85	CCl3 sym stretch
653	A"	0.00	0.82	C(1) oop bend
721	A'	148.17	10.41	CCl3 asym stretch
743	A"	313.02	6.11	CCl3 asym stretch
854	A'	400.04	6.10	Cl(1)-C(1) stretch
916	A'	203.31	6.61	O(2)-C(2) stretch
989	A'	681.27	4.45	C(1)-O(2) stretch
1818	A'	260.15	7.06	C(1)=O(1) stretch

[1] irreducible representation in C_s symmetry.
[2] Cl(1)–C(1)(=O(1))–O(2)–C(2)–Cl(2ip)–Cl(3oop)(Cl3’oop) (ip = in-plane, oop = out-of-plane).
Note: translations and librations have been omitted, i.e. internal modes in C_s symmetry only.

Table S11: Calculated spectra of triphosgene with idealized C_{2v} symmetry.

Transition energy / cm^{-1}	Rep.[1]	Infrared intensity / km mol^{-1}	Raman intensity / $Å^4$ amu^{-1}	Assignment[2]
25	A2	0.00	0.16	CCl3 antiphase torsion
42	B1	0.00	0.14	CCl3 rock
78	A1	0.15	0.41	CCl3 rock
102	A2	0.00	2.34	CCl3 rock
118	B1	0.00	0.92	O(2)C(2) + C(2)O(3) ip torsion
149	B2	0.15	1.68	CCl3 rock
165	A1	0.07	3.11	O(1)C(2)O(3) bend
225	A1	0.32	3.94	CCl3 asym bend
235	B1	0.02	1.31	CCl3 asym bend
243	A2	0.00	0.76	CCl3 asym bend
255	B2	1.40	0.06	CCl3 asym bend
311	B2	0.01	1.91	C(1)-O(1)-C(2) + C(2)-O(2)-C(3) out-of-phase bend
336	A1	9.64	10.89	C(1)-O(1)-C(2) + C(2)-O(2)-C(3) in-phase bend
345	B2	0.15	2.64	CCl3 oop sym bend
373	B1	7.74	2.78	Skeletal deformation
393	A1	0.00	34.80	CCl3 in-phase sym bend
393	A2	0.00	1.28	Skeletal deformation
506	B2	70.03	0.09	CCl3 out-of-phase sym stretch
645	A1	87.93	11.75	C=O ip bend
645	B2	16.18	0.58	CCl3 in-phase sym stretch
725	B1	32.33	0.24	C(2) out-of-plane deformation
743	A2	0.00	2.63	CCl3 asym stretch
753	B1	544.98	3.99	CCl3 asym stretch
773	A1	198.38	5.89	CCl3 asym stretch
829	B2	0.03	3.80	CCl3 asym stretch
888	B2	1231.39	0.42	C(1)-O(1) + O(3)-C(3) out-of-phase stretch
923	A1	191.27	11.40	C(1)-O(1) + O(3)-C(3) in-phase stretch
1060	A1	9.38	6.00	O(1)-C(2) + C(2)-O(3) in-phase stretch
1120	B2	677.78	0.62	O(1)-C(2) + C(2)-O(3) out-of-phase stretch
1817	A1	230.39	3.85	C(2)=O(2) stretch

[1] irreducible representation in C_{2v} symmetry.

[2] Cl(2oop)(Cl2'oop)Cl(1ip)–C(1)–O(1)–C(2)(=O(2))–O(3)–C(3)–Cl(3ip)–Cl(4oop)(Cl4'oop)

Note: translations and librations have been omitted, i.e. internal modes in C_{2v} symmetry only.

6. References

[1] V. B. Arce, C. O. Della Védova, A. J. Downs, S. Parsons, R. M. Romano, *J. Org. Chem.* **2006**, *71*, 3423.

[2] W. G. Fateley, F. R. Dollish, N. T. McDevitt, F. F. Bentley, *Infrared and Raman selection rules for molecular and lattice vibrations: the correlation method*, Wiley, New York, **1972**.
